# Genetically Encoded Protein Thermometer Enables Precise Electrothermal Control of Transgene Expression

**DOI:** 10.1002/advs.202101813

**Published:** 2021-09-08

**Authors:** Bozhidar‐Adrian Stefanov, Ana P. Teixeira, Maysam Mansouri, Adrian Bertschi, Krzysztof Krawczyk, Ghislaine Charpin‐El Hamri, Shuai Xue, Martin Fussenegger

**Affiliations:** ^1^ ETH Zürich Department of Biosystems Science and Engineering Mattenstrasse 26 Basel 4058 Switzerland; ^2^ Département Génie Biologique Institut Universitaire de Technologie Villeurbanne Cedex F‐69622 France; ^3^ University of Basel Faculty of Life Science Basel 4056 Switzerland

**Keywords:** diabetes, gene regulation, gene switches, synthetic biology, temperature

## Abstract

Body temperature is maintained at around 37 °C in humans, but may rise to 40 °C or more during high‐grade fever, which occurs in most adults who are seriously ill. However, endogenous temperature sensors, such as ion channels and heat‐shock promoters, are fully activated only at noxious temperatures above this range, making them unsuitable for medical applications. Here, a genetically encoded protein thermometer (human enhanced gene activation thermometer; HEAT) is designed that can trigger transgene expression in the range of 37–40 °C by linking a mutant coiled‐coil temperature‐responsive protein sensor to a synthetic transcription factor. To validate the construct, a HEAT‐transgenic monoclonal human cell line, FeverSense, is generated and it is confirmed that it works as a fever sensor that can temperature‐ and exposure‐time‐dependently trigger reporter gene expression in vitro and in vivo. For translational proof of concept, microencapsulated designer cells stably expressing a HEAT‐controlled insulin production cassette in a mouse model of type‐1 diabetes are subcutaneously implanted and topical heating patches are used to apply heat corresponding to a warm sensation in humans. Insulin release is induced, restoring normoglycemia. Thus, HEAT appears to be suitable for practical electrothermal control of cell‐based therapy, and may also have potential for next‐generation treatment of fever‐associated medical conditions.

## Graphical abstract

Existing cellular temperature sensors, such as ion channels and heat‐shock promoters, are fully activated only by heat at a painful level, making them unsuitable for bioengineering‐inspired medical applications. Here, a temperature‐sensing protein is developed functional in the physiological range of 37–40 °C. This enables precise electrothermal control of cellular therapies in living animals using a simple, programmable heatpad.

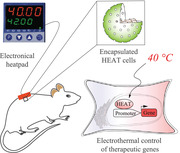

## 1 Introduction

Since metabolic reaction networks are highly temperature sensitive, most living systems have evolved to function in ecosystems with a narrow temperature range, while having the ability to survive short‐term environmental temperature fluctuations.^[^ ^1^, ^2^ ^]^ Endothermic species such as humans actively control their body temperature to remain within a very narrow range, enabling them to be largely independent of daily and seasonal temperature shifts.^[^ ^3^, ^4^ ^]^ For this purpose, the hypothalamus acts as central thermostat by integrating temperature inputs from cold‐ and heat‐sensitive neuronal fibers that collect information from thermal sensors, such as cold‐activated (TRPA1, TRPM8) and heat‐activated (TRPV1, TRPM2, TRPM3) channels,^[^ ^5^, ^6^, ^7^, ^8^ ^]^ across the entire body. The hypothalamus adjusts heat production through muscle contraction (shivering) or heat dissipation (perspiration) as required via a range of hormone‐based closed‐loop control circuits.^[^ ^3^, ^6^ ^]^ The hypothalamus also actively increases the body temperature as a defence mechanism to contain viral, bacterial and parasitic infections, such as influenza, meningitis, COVID‐19 and malaria^[^ ^9^, ^10^, ^11^, ^12^ ^]^ a process known as pyroxia or fever. This transient increase of the body's homothermic temperature by a few degrees Celsius is one of the most common medical signs and occurs in about 75% of adults who are seriously ill.^[^ ^13^ ^]^ At the cellular level, thermal emergency situations are attenuated by a panoply of chaperone molecules^[^ ^14^, ^15^ ^]^ and heat‐shock promoters, which manage unfolded protein responses in a feed‐forward control manner.^[^ ^14^, ^16^ ^]^

In contrast, microorganisms are unable to actively control their temperature, but have evolved to survive in extreme‐temperature ecosystems and to adapt rapidly to temperature shifts.^[^ ^1^, ^17^, ^18^ ^]^ They employ a variety of temperature‐sensitive molecular switches, including RNA thermosensors^[^ ^19^ ^]^ and transcription factors,^[^ ^17^, ^18^, ^20^ ^]^ to respond to changes of temperature via altered cis‐regulatory base‐pairing and dimerization‐dependent DNA‐binding, respectively. This ability is particularly prominent for human pathogens, which shuttle between cooler environments and their warmer host habitats.^[^ ^17^, ^18^, ^20^ ^]^ For example, the intracellular pathogen *Salmonella typhimurium* immediately recognizes the body temperature of its host upon infection and adapts by the expression of virulence factors that promote its survival and persistence inside human cells.^[^ ^18^ ^]^ The cytoplasmic protein TlpA encoded on a virulence plasmid of *S. typhimurium* was one of the first documented temperature‐sensing gene regulators.^[^ ^18^, ^21^ ^]^ TlpA contains a C‐terminal *α*‐helical coiled‐coil motif and a sequence‐specific DNA‐binding domain acting as an autoregulatory repressor.^[^ ^18^ ^]^ At cooler environmental temperatures TlpA forms a dimeric folded coiled‐coil conformation that is able to bind and repress the *tlpA* promoter. At warmer intracellular temperatures around 37 °C, TlpA adopts a nonfunctional unfolded monomeric form which derepresses *tlpA*.^[^ ^18^, ^22^ ^]^ TlpA has recently been successfully engineered to control green‐fluorescent protein (GFP) expression in *Escherichia coli* maintained in culture^[^ ^23^ ^]^ or injected into nude mice.^[^ ^24^ ^]^ Although TlpA‐GFP fusion proteins could be used to visualize subcellular thermoregulation in living mammalian cells, attempts to achieve TlpA‐mediated temperature‐responsive gene expression have been unsuccessful.^[^ ^23^, ^25^ ^]^ In addition, previous studies have found that low‐temperature induction systems based on viral replicons^[^ ^26^ ^]^ or the bacterial RheA repressor^[^ ^27^ ^]^ are not functional in mammals. Even the cold‐sensation channel hTRPM8 was insensitive in mice kept at low ambient temperatures, since these warm‐blooded animals can maintain their body temperature in cold environments.^[^ ^28^ ^]^ Furthermore, although native heat‐shock promoters appear to be functional in mammalian cells^[^ ^29^ ^]^ as well as in mice,^[^ ^30^, ^31^ ^]^ their activation requires noxious temperatures associated with tissue damage upon prolonged exposure,^[^ ^32^ ^]^ and their use involves sophisticated equipment such as magnetic resonance imaging (MRI)‐guided high‐intensity‐focused ultrasound^[^ ^30^ ^]^ or magnetic nanoparticles,^[^ ^31^ ^]^ which are suspected to elicit adverse health effects.^[^ ^33^ ^]^ Thus, the quest for an orthogonal, genetically encoded mammalian protein thermometer capable of programming transgene expression in human cells is ongoing.

To meet this need, we have designed a human enhanced gene activation thermometer (HEAT) that enables precise, robust and reversible temperature‐adjustable gene expression within the pathophysiologically relevant temperature range of 37–40 °C. Using an in vivo mouse model of type‐1 diabetes, we confirmed that subcutaneously implanted microencapsulated designer cells stably expressing a HEAT‐controlled insulin production cassette can restore normoglycemia in response to topical warming with electrothermal patches.

## 2 Results

### 2.1 Thermocontrol by Endogenous Heat‐Sensing Mechanisms

Inspired by the use of the human cold‐sensitive channel TRPM8 to program successful treatment of experimental muscle atrophy in response to cooling sensation,^[^ ^28^ ^]^ we tested the mammalian heat‐sensitive channels TRPV1 and TRPV4 for temperature‐responsive activation of synthetic calcium‐dependent signaling cascades (Figure S1a, Supporting Information). However, constitutively expressed TRPV1 or TRPV4 showed only modest fold induction, even at 44 °C, which represents a noxious temperature^[^ ^32^, ^34^ ^]^ (Figure S1b, Supporting Information). With a goal to achieve innoxious temperature induction, we placed either the native human HSP70B’ promoter sequence or the tandem HSPA1A‐derived heat‐shock elements upstream of a minimal eukaryotic promoter following established in vitro designs.^[^ ^14^, ^35^ ^]^ Both synthetic heat‐shock promoters were strongly induced at 44 °C, but showed only marginal induction below 43 °C (Figure S1c,d, Supporting Information). Wild‐type mice, subcutaneously implanted with engineered cells for heat‐shock promoter‐driven SEAP (human placental secreted alkaline phosphatase) expression, whose skin surface temperature was increased to 44 °C by infrared illumination or topical heat patches, showed only modestly increased blood SEAP levels (Figure S1e, Supporting Information), suggesting that the animals’ homeostatic control compensated for external heating of the skin. These results suggest that orthogonal temperature‐inducible transgene regulation remains challenging in living animals and likely requires genetically encoded temperature sensors to operate much closer to the homeostatic temperature.^[^ ^36^, ^37^ ^]^

### 2.2 Toward the Design of an Orthogonal Mammalian Protein Thermometer

**Figure 1:**
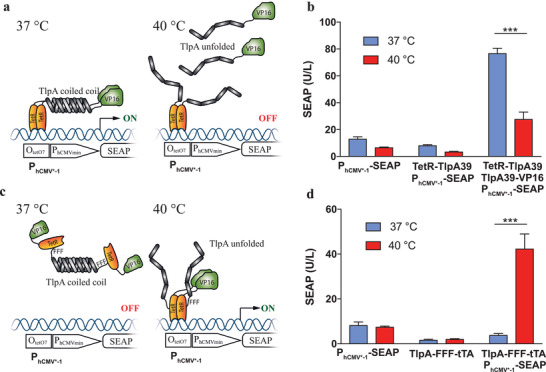
Engineering temperature‐controlled gene switches in mammalian cells. a) Design of a temperature repressible system. Formation of a coiled‐coil dimer by TlpA_39_ at 37 °C reconstitutes the TetR‐VP16 (tTA) transactivator from TlpA_39_‐VP16 (pAna117) and TetR‐TlpA_39_ (pAna124), which induces SEAP reporter gene expression from a P_hCMV*‐1_ promoter consisting of an O_TetO7_ sequence upstream to a minimal P_hCMVmin_ promoter. Temperature‐induced unfolding of the TlpA_39_ dimer results in dissociation of the transactivation complex and inhibits reporter gene expression. b) Transfection of cells with the reporter plasmid alone or together with TetR‐TlpA_39_ (pAna124) is not sufficient to induce gene expression. When TlpA_39_‐VP16 (pAna117) is co‐expressed, SEAP expression occurs, and the expressed amount is decreased in cells exposed to 40 °C. c) Design of a temperature inducible system. Formation of a coiled‐coil dimer by TlpA_39_ at 37 °C blocks transcriptional activation by a TlpA_39_‐FFF‐TetR‐VP16 fusion complex from a P_hCMV*‐1_ promoter. Heating induces the unfolding of TlpA_39_ dimers, converting the transactivation complex into its active form and inducing SEAP gene expression. c) The P_hCMV*‐1_ reporter or the TlpA_39_‐FFF‐TetR‐VP16 (P_hCMV_‐TlpA39‐FFF‐TetR‐VP16‐pA_bGH_, pBS707) transactivation complex alone is not sufficient to activate gene expression. Co‐transfection of both vectors results in temperature inducible SEAP reporter secretion in the supernatant upon incubation at 40 °C. Data in (b) and (d) are mean ± SD of *n* = 3 biologically independent samples, representative of *n* = 3 independent experiments. Statistical analysis was done with a two‐tailed *t*‐test: *** *p* < 0.001.

Since endogenous thermosensors such as channels and heat‐shock promoters did not provide acceptable temperature sensitivity, we considered other avenues to engineer an orthogonal mammalian protein thermometer. Capitalizing on the heat‐inducible DNA‐binding ability of TlpA, which contributes to infection‐associated temperature adaptation of *S. typhimurium*, we fused a modified TlpA variant (TlpA_39_;^[^ ^24^ ^]^) to the VP16 transactivation domain of the *Herpes simplex* virus. This resulted in a synthetic mammalian transcription factor (pAna117, P_hCMV_‐TlpA_39_‐VP16‐pA_bGH_) binding to chimeric promoters containing a TlpA‐specific operator sequence fused to a minimal promoter (pAna115, O_TlpA_‐P_min_‐SEAP‐pA_bGH_) in a temperature‐repressible manner. However, this classical gene switch topology was unable to activate SEAP expression beyond background in human cells (37 °C, 1.6 ± 0.3 U/L; 40 °C, 2.0 ± 0.3 U/L), so we next tested a split transcription factor design. The *E. coli* tetracycline‐dependent repressor (TetR) was C‐terminally fused (pAna126, P_hCMV_‐TetR‐TlpA_39_‐pA_bGH_) and VP16 was N‐terminally fused to TlpA (pAna117, P_hCMV_‐TlpA_39_‐VP16‐pA_bGH_). Dimerization of TlpA's coiled‐coil domains at 37 °C reconstitutes a tetracycline‐dependent transactivator (tTA) variant (TetR‐TlpA_39_‐TlpA_39_‐VP16), which binds to and activates standard tTA‐dependent tetracycline‐responsive promoters (P_hCMV*‐1_) (**Figure** **1**a,b). At 40 °C, the coiled‐coil domains unfold and the tTA variant splits, thereby shutting off P_hCMV*‐1_‐driven target‐gene expression (Figure 1). Although this prototypic mammalian thermometer design was in principle responsive between 37 °C and 40 °C, its overall leakiness was high, limiting the induction‐fold to 2.5 (Figure 1b). In addition, this OFF‐type device can only be shut down at elevated temperatures, which would be problematic for potential clinical applications.

### 2.3 Design and Validation of the Human Enhanced Gene Activation Thermometer (HEAT)

Instead of reconstituting a synthetic transcription factor via TlpA‐derived coiled‐coil domain‐mediated heterodimerization in a high‐temperature‐repressible manner, we next directly linked a single TlpA domain C‐terminally to the tetracycline‐dependent transactivator (tTA) via a triple VP16‐derived F‐type minimal transactivation domain (pBS707, P_hCMV_‐TlpA_39_‐FFF‐TetR‐VP16‐pA_bGH_) (Figure 1c). In this system, the TlpA coiled‐coil domains lock tTA in an inactive homodimerized configuration at 37 °C (Figure 1c). At higher temperatures, however, the TlpA moiety adopts an unfolded monomeric state, releasing functional tTA, which can bind to and activate classical tTA‐dependent tetracycline‐responsive promoters (Figure 1c). Indeed, as expected, the TlpA‐FFF‐tTA thermometer remained inactive at 37 °C and provided fully induced P_hCMV*‐1_‐driven SEAP expression in human HEK‐293 cells at 40 °C (Figure 1d). While the viability of isogenic cultures constitutively expressing SEAP was comparable at 37 °C and at 40 °C, the overall viable cell count was lower at 40 °C, indicating that proliferation of human HEK‐293 cells was lower at elevated temperature (Figure S2a,b, Supporting Information). Despite this, the TlpA‐FFF‐tTA‐mediated P_hCMV*‐1_ was repressed at 37 °C but induced at 40 °C, reaching SEAP values comparable to those driven by either native tTA or the isogenic constitutive promoter P_hCMV_ (Figure S2c, Supporting Information).

### 2.4 Detailed Characterization of HEAT Performance In Vitro

Performance profiling in different cell types confirmed 37 °C‐OFF, 40 °C‐ON thermocontrol by HEAT in a variety of rodent and human cell lines, suggesting that HEAT would be broadly applicable for basic and translational research, and would be compatible with biopharmaceutical manufacturing (CHO‐K1, BHK‐21, HEK‐293) as well as major tissues, including liver (Hep G2), colon (Caco‐2), muscle (C2C12) and connective tissue (HT‐1080) (Figure S3, Supporting Information). In particular, HEAT is able to program gene expression in human mesenchymal stem cells (hMSCs) and also in patient‐derived induced pluripotent stem cells (iPSCs), which are currently in the limelight as candidates for the next wave of cell‐based therapies.^[^ ^38^, ^39^ ^]^ Importantly, HEAT was specifically temperature‐inducible, both by exposure to 40 °C and by a heat‐shock pulse, and was orthogonal to other physiological inducers (Figure S4a,b, Supporting Information). In comparison, the best‐performing heat‐shock promoter did not show significant induction upon incubation at 40 °C and was activated by the anti‐cancer medication luminespib (Figure S4c,d, Supporting Information).

**Figure 2:**
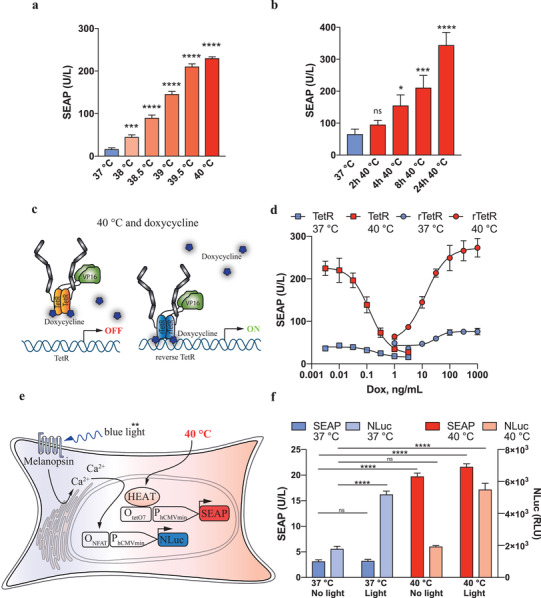
Characterization of the human enhanced gene activation thermometer (HEAT) as a genetically encoded temperature‐operated controller for cellular therapy. a) The temperature–response relationship of HEAT (pBS707) for transcription from a P_hCMV*‐1_‐controlled SEAP expression vector in transfected HEK‐293 cells was characterized by incubating the cells at the indicated temperatures from 37 to 40 °C (in the physiological fever range). b) The time–response relationship of HEAT (pBS707) for transcription from a P_hCMV*‐1_ controlled SEAP reporter vector in transfected HEK‐293 cells was characterized by incubating the cells for the indicated duration at 40 °C and then returning them to 37 °C for the rest of the 24 h period. c) Integration of a small‐molecule safety switch into the HEAT system is enabled by the TetR DNA binding domain, which loses affinity for its recognition sequence upon binding of doxycycline. This prevents the activation of target genes even at 40 °C. Exchange of TetR for the reverse TetR DNA binding domain results in an AND‐gate logic circuit where both the temperature stimulus and doxycycline are necessary for the activation of target genes. d) Doxycycline dose‐dependent relationship of SEAP reporter gene expression at 37 and 40 °C from cells transfected with a P_hCMV*‐1_ controlled reporter vector and HEAT (P_hCMV_‐TlpA39‐FFF‐TetR‐VP16‐pA_bGH_, pBS707) or a TlpA_39_‐rtTA (P_hCMV_‐TlpA39‐FFF‐rTetR‐VP16‐pA_bGH_, pAB659; rTetR – reverse TetR) temperature‐inducible transactivator. e) Multiplexing of dual physical stimuli through the blue‐light‐responsive melanopsin protein rerouted into a reporter for intracellular calcium signaling (O_NFAT_) and the temperature‐inducible transcriptional factor directly activating gene expression from a promoter flanked by the TetR recognition sequence O_tetO7_. f) Multiplexing of blue light‐inducible nanoluciferase (NLuc) and warmth‐inducible SEAP reporter gene expression. Cells transgenic for both melanopsin receptor (pHY42) and HEAT (P_hCMV_‐TlpA39‐TetR‐VP16‐pA_bGH_, pBS707) were stimulated with blue light and warmth (40 °C) separately or with both traceless input signals simultaneously. Blue light‐inducible NLuc and warmth‐inducible SEAP reporter proteins were measured in the culture supernatants. Data are mean ± SD of *n* = 3 biologically independent samples, representative of three independent experiments. Statistical analysis of multiple groups was done with one‐way ANOVA, compared to the control group (37 °C): *p < 0.05, **p < 0.01, ***p < 0.001, **** *p* < 0.0001.

HEAT showed highly temperature‐sensitive induction of SEAP expression in the pathophysiological range of 37–40°C (Figure**2a** and distinctive temperature‐programmable expression kinetics (Figure 2b, Figure S5, Supporting Information). Additionally, HEAT fully retained the tetracycline responsiveness of its tTA moiety, as SEAP expression by HEAT‐transgenic cells could be dose‐dependently shut down at 40 °C by exposure to increasing concentrations of the clinically licensed antibiotic doxycycline (Figure 2c,d). This demonstrates the dual‐input responsiveness of the HEAT device to both small‐molecular and physical cues and furthermore suggests that doxycycline interventions could serve both as an additional layer of adjustability and as a safety trigger to transiently override the thermometer in emergency situations (Figure 2d). These characteristics would be important assets in potential future clinical applications. Further, when we replaced HEAT's tTA domain with its reverse tTA (rtTA) variant (pAB659, P_hCMV_‐TlpA_39_‐FFF‐rTetR‐VP16‐pA_bGH_), HEAT provided AND‐gate‐type temperature *and* doxycycline inducibility (Figure 2d). HEAT was also fully compatible with the melanopsin‐based light switch (Figure 2e,f),^[^ ^40^ ^]^ and could be multiplexed to enable independent, interference‐free, traceless control of two sets of transgenes using nonmolecular physical cues (Figure 2f).

### 2.5 HEAT Acts as a Fever Thermometer in Mice

**Figure 3:**
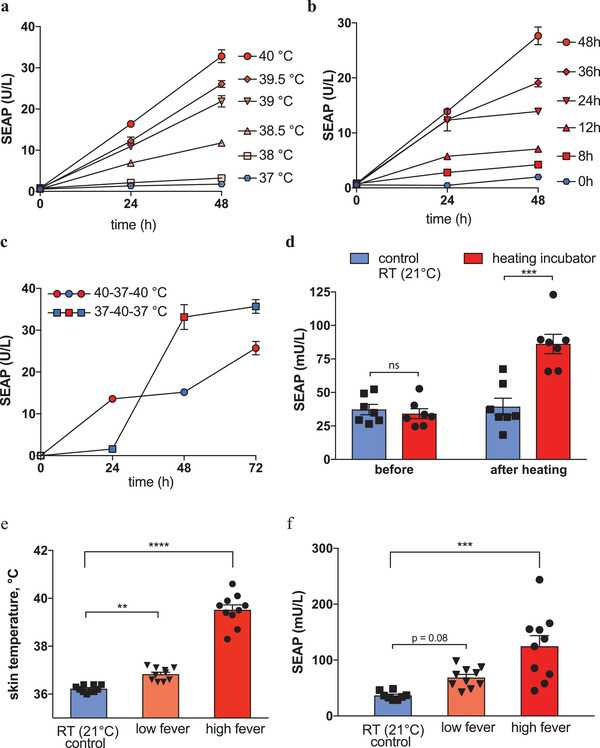
Fever‐sensing cells and their performance in murine models of elevated body temperature. a) Temperature–response relationship for FeverSense cells, which stably express TlpA_39_‐FFF‐tTA (P_hEF1*α*_‐TlpA_39_‐FFF‐TetR‐VP16‐pA_bGH_, pBS786) and SEAP from a P_hCMV*‐1_ promoter (pBS828). b) Time‐dependent induction of SEAP expression in FeverSense cells over a fixed period of 48 h. At the start of the 48 h measurement period, cells were subjected to a 40 °C stimulation of the indicated duration and were then cultured at 37 °C for the remaining time. c) Characterization of the reversibility of SEAP reporter gene expression by FeverSense cells stably expressing TlpA_39_‐FFF‐tTA (P_hEF1*α*_‐TlpA_39_‐TetR‐VP16‐pA_bGH_, pBS786) and a SEAP reporter gene expression vector controlled by a P_hCMV*‐1_ promoter. Cells were subjected to two temperature profiles consisting of incubation periods at each temperature: 40–37–40 °C or 37–40–37 °C, and SEAP accumulation was quantified in the culture supernatant every 24 h. d) Subcutaneous implant containing FeverSense designer cells can differentiate between normal and elevated body temperatures. Microencapsulated engineered cells were injected into the back of Swiss albino mice. SEAP reporter protein was quantified in serum samples (*n* = 7 per group, 1 outlier excluded per group) collected before and after housing one of the groups at 36 °C for 48 h. e) Skin surface temperature measurement of c57bl/6 mice at the end of a representative 2 h cycle at 39.5 °C in a model consisting of 4 cycles in 9 h d^−1^ to simulate high fever episodes. f) FeverSense cells differentiate between homeostatic temperature, prolonged low‐grade fever, and high‐grade fever models in c57bl/6 mice. Microencapsulated designer cells were injected subcutaneously on the back of the mice, which were then either maintained at 21 °C as a room temperature control (*n* = 8), maintained at 36 °C as a prolonged low fever simulation (*n* = 10), or placed at 39.5 °C for four cycles of 2 h interspaced by 15 min recovery periods for a total of 9 h to simulate severe fever episodes (*n* = 10). Data in (a)–(c) are mean ± SD of *n* = 3 biologically independent samples, representative of *n* = 3 independent experiments. Small error bars covered by the symbol are not shown. Data in (d)–(f) are shown as bar graphs of mean ± SEM, overlaid with the individual data points. Statistical analysis in (d) was done with a two‐tailed *t*‐test and in (e) and (f) with one‐way ANOVA for multiple comparisons: *p < 0.05, **p < 0.01, ***p < 0.001, ****p < 0.0001.

In humans, the body's temperature set point is around 37 °C and fevers are not typically higher than 40.5 °C.^[^ ^9^, ^13^ ^]^ Therefore, a molecular fever sensor has to react to quite small changes in body temperature (Figure 2a) and provide precise temperature‐responsive induction kinetics (Figure 2b). To validate HEAT's fever response in vitro, we generated HEAT‐transgenic monoclonal human cell lines (Figure S6, Supporting Information). The best‐in‐class temperature‐inducible clonal cell line, FeverSense, showed finely graded temperature induction profiles between 37 and 40 °C with sub‐degree precision **3a**. Also, temperature‐inducible SEAP induction kinetics could be precisely programmed by employing different high‐temperature exposure times, indicating that FeverSense would be able to sense and react to the severity as well as the duration of a febrile state (Figure 3b). In addition, the FeverSense actuation profile was rapidly reversible, showing baseline SEAP expression upon cooling from 40 to 37 °C. (Figure 3c).

In contrast to humans, infectious febrile responses in mice are of shorter duration and involve a lower temperature rise,^[^ ^41^ ^]^ which sets additional performance challenges for the HEAT device in an experimental model.^[^ ^42^ ^]^ To validate HEAT's fever response in vivo, we implanted microencapsulated FeverSense cells into mice and either maintained the animals at room temperature (21 °C) or exposed them to an ambient temperature of 36 °C, which increases their core body temperature, thereby simulating fever.^[^ ^42^, ^43^, ^44^ ^]^ Indeed, while animals kept at room temperature maintained a standard homeostatic body temperature of around 36 °C and showed only basal SEAP expression, mice kept in the hot environment exhibited a body temperature above 38 °C, corresponding to a mild fever state,^[^ ^43^ ^]^ and showed increased circulating SEAP level in their bloodstream, demonstrating that HEAT‐transgenic FeverSense cells were able to sense and react to fever (Figure 3d).

Next, to simulate a high‐grade fever, we repeatedly exposed the animals to 39.5 °C for short periods,^[^ ^45^ ^]^ driving their skin temperatures above 39 °C (Figure 3e). This treatment increased the blood SEAP levels compared to those of animals with a body temperature of 36 °C or 38 °C (Figure 3f). Overall, these results show that HEAT was able to detect and differentiate between different body temperatures in the range from normal to high‐grade fever, and was able to program a proxy biopharmaceutical response accordingly. To provide additional insight into the clinical potential of the system, we demonstrated that HEAT‐transfected bone‐marrow‐derived hMSCs are not affected by the increased temperature (Figure S7a, Supporting Information) and that the system is functional upon stable integration of the components into the genome of these cells (Figure S7b,c, Supporting Information). When encapsulated in a protective alginate matrix the obtained induction for the stable hMSCs was comparable to that of the FeverSense cells (Figure S8a,b, Supporting Information). Furthermore, subcutaneous implantation in mice enabled control over gene expression by using an electronic heatpad (Figure S9, Supporting Information).

### 2.6 Noninvasive Heat‐Patch‐Controlled Insulin Release Restores Glucose Homeostasis in Experimental Type‐1 Diabetes

**Figure 4:**
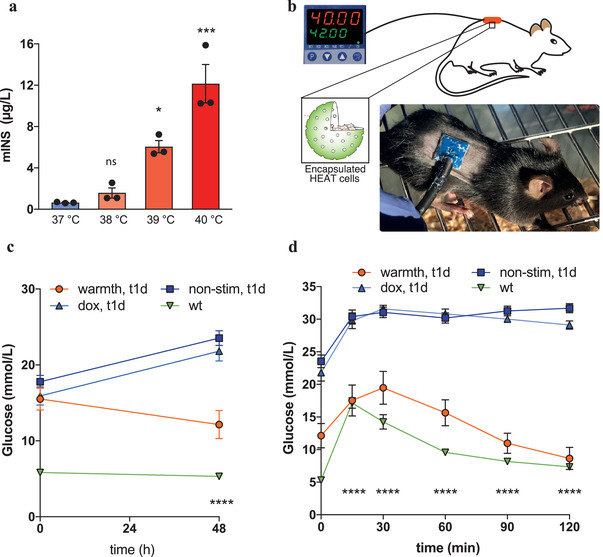
Noninvasive extracorporeal‐temperature‐regulated secretion of insulin for management of type‐1 diabetes. a) Temperature‐regulated insulin release from alginate‐PLL‐encapsulated designer cells stably expressing HEAT (P_hPGK_‐TlpA_39_‐FFF‐TetR‐VP16‐pA_bGH_, pBS830) and a P_hCMV*‐1_‐controlled optimized mouse insulin production vector. The encapsulated cells were stimulated for 48 h with the indicated temperatures, and insulin concentration was profiled in the culture supernatants. Data are mean values shown as a bar graph ± SEM overlaid with individual data points. b) Scheme of extracorporeal electrothermal control of insulin delivery in mice. A 1 cm^2^ heating plate is positioned over a subcutaneous implant consisting of alginate‐PLL‐encapsulated engineered cells. An electronic microcontroller is used to regulate the temperature of the heating plate, and thus the temperature of the encapsulated cells and the released dose of insulin. An area of 4 cm^2^ on the back of mice was shaved for subcutaneous implantation of engineered cells. The heating patch was fixed with clinically approved elastic compression band for the duration of the treatment. c) Fasting glycemia of mice was recorded before implantation of therapeutic cells and after 2 d of treatment with a total of 8 h stimulation per day (warmth, t1d). Control groups were wild‐type mice (wt), type‐1 diabetic mice with nonstimulated implants (nonstim, t1d), and type‐1 diabetic mice with nonstimulated implants receiving 0.5 mg doxycycline in 250 µL oral gavage to simulate a switched‐off implant (dox, t1d). d) Glucose tolerance test (GTT) in mice fasted for 8 h, 2 d after the start of treatment with the therapeutic designer cells. Warmth stimulation of the designer cell implant was done for 8 h before conducting the GTT. The experimental groups were wild‐type mice (wt), type‐1 diabetic mice with warmth‐treatment‐stimulated implants (warmth, t1d), nonstimulated implants (nonstim, t1d), or with nonstimulated implants and receiving 0.5 mg doxycycline in 250 µL oral gavage to simulate a switched‐off implant (dox, t1d). The measured values were capped at 33.3 × 10^−9^ m (600 mg dL^−1^) glucose. Data in (a) are shown as a bar graph of mean ± SEM, overlaid with the individual data points. In (c) and (d), data are mean ± SEM for 8 mice (*n* = 8). Statistical analysis was done with one‐way ANOVA for multiple comparisons: * *p* < 0.05, **p < 0.01, ***p < 0.001, ****p < 0.0001. In (c) and (d), the significance of the mean difference of stimulated (warmth, t1d) versus unstimulated (control, t1d) is indicated.

In order to further expand the potential applications of HEAT, we considered the fact that touching human skin with objects having a temperature between 39 and 40 °C triggers a pleasant sensation of warmth.^[^ ^46^ ^]^ Since this is within the working range of HEAT, we examined whether warming local skin tissue to this temperature with an electrothermal skin patch would be effective for percutaneous HEAT‐mediated remote‐control of biopharmaceutical protein production, release and systemic delivery by designer cells. Electrothermal skin patches are particularly attractive for therapeutic interventions using cell‐based therapies, as they enable traceless control in the absence of any molecular cues or co‐factors, can be discretely and comfortably worn during therapy, and provide a direct link between wearable electronics and therapeutic gene expression. Therefore, as a translational proof‐of‐concept, we first linked HEAT sensing to expression of insulin and produced stable monoclonal human cell lines that provide temperature‐actuated release of this peptide hormone (Figure S10, Supporting Information). The best‐in‐class cell line showed precise temperature‐adjustable insulin release in the warm‐sensation range, even when microencapsulated in clinically licensed semi‐permeable alginate to protect them from the mouse immune system (**Figure** **4**a).

To enable precise transdermal control, we designed a companion electrothermal skin patch that was operated by a compact electronic controller (Figure 4b). With its skin‐contact surface of 1 cm^2^ and its small and light‐weight (0.5 g) design, the patch could be easily strapped to the skin immediately above a subcutaneously placed implant (Figure 4b). Type‐1‐diabetic mice subcutaneously implanted with microencapsulated HEAT‐transgenic insulin‐producing cells and equipped with heating patches showed continued hyperglycemia without any improvement of their diabetic state when the patch remained switched off, suggesting that the engineered cells do not show any physiologically relevant leakiness (Figure 4c). However, fasting blood‐glucose levels rapidly decreased upon activation of the heating patch, confirming the effectiveness of warmth‐mediated remote‐control of insulin release by the implanted cells (Figure 4c). The HEAT device was not only able to restore normoglycemia in the type‐1‐diabetic mice, but also provided sufficient insulin to rapidly attenuate postprandial blood‐glucose spikes simulated in glucose‐tolerance tests (Figure 4d). In contrast, untreated mice and treated animals with the heating patch switched off retained extreme hyperglycemia. These results indicate that the HEAT‐based traceless electrothermal pecutaneous programming of cell implants is robust and reliable to correct one of the most difficult‐to‐control endocrine conditions.

## 3 Discussion

Next‐generation cell‐based therapies require sophisticated gene switches programming precise, adjustable and reversible trigger‐inducible therapeutic gene expression in order to deliver therapeutic proteins at effective doses in a timely manner.^[^ ^39^ ^]^ Many trigger‐inducible gene switches responsive to small‐molecular compounds have been developed and used to modulate the activity of synthetic transcription factors,^[^ ^47^, ^48^ ^]^ cell‐surface receptors and membrane channels,^[^ ^28^ ^]^ or to activate chimeric promoters^[^ ^48^ ^]^ and synthetic signaling cascades^[^ ^49^ ^]^ in order to produce and release therapeutic proteins.^[^ ^39^, ^48^ ^]^ Trigger compounds include drugs,^[^ ^50^ ^]^ anti‐infectives,^[^ ^47^ ^]^ vitamins,^[^ ^51^ ^]^ food additives^[^ ^52^ ^]^ and metabolites.^[^ ^48^ ^]^ But, despite these advances, issues of cytotoxicity, pharmacodynamics, bioavailability, accumulation, and pleiotropism of the trigger compounds and co‐factors for a long time remained as major challenges impeding real‐world medical applications.^[^ ^53^ ^]^ These challenges were largely overcome with the advent of traceless control inputs such as light,^[^ ^40^ ^]^ magnetic fields,^[^ ^31^, ^54^ ^]^ radio waves^[^ ^55^, ^56^ ^]^ and electric currents.^[^ ^57^ ^]^ Nevertheless, many of these traceless control circuits require sophisticated stimulation hardware, complex chemical co‐factors or harmful inorganic nanoparticles.^[^ ^31^, ^33^ ^]^ Also, traceless control systems are responsive to the orthogonal input, but are typically unable to sense and react to endogenous signals. In the present work, we have engineered HEAT as a temperature sensitive control device working in the physiological range; it can respond either to endogenous fever or to external heating at a level corresponding to a sensation of warmth in humans. HEAT offers excellent control parameters, providing robust, reversible and precisely adjustable trigger‐inducible ON‐type expression profiles, and does not require any co‐factor or sophisticated equipment. As a generalized expression system, HEAT should be applicable for improved control of multiple diseases by the therapeutic delivery of hormones (such as desmopressin for the treatment of nocturia), peptides (such as glp1 for type 2 diabetes) or antibodies (such as anti‐IL6R in inflammatory conditions). From the viewpoint of potential clinical applications, HEAT actuation can be fine‐tuned or completely overridden in an emergency by clinically licensed tetracycline drugs. Owing to the bacterial origin of its components, it might be necessary to incorporate an immunological mask for this transcriptional factor^[^ ^58^ ^]^ in the case of applications where no protective barrier would shield the engineered cells.

Our translational proof‐of‐concept study demonstrated that simple electrothermal induction using topical skin patches was effective to percutaneously program insulin release by subcutaneously implanted insulin‐releasing cells and to restore normoglycemia in experimental type‐1 diabetes in mice. We chose this model because type‐1 diabetes is one of the most common and difficult‐to‐control metabolic disorders, with dramatically increasing prevalence worldwide.^[^ ^59^ ^]^ Importantly, HEAT was able not only to restore fasting blood‐glucose levels to the normal range, but also to attenuate short‐term postprandial blood‐glucose spikes, restoring near wild‐type kinetics. With wearable electronic devices profiling blood‐glucose levels noninvasively on the horizon,^[^ ^60^ ^]^ noninvasive closed‐loop control of diabetes by wearable electronic devices combining optogenetics and thermoelectronics seems to be within reach. Moreover, as HEAT is compatible with optogenetic devices, it could find application for interference‐free, independent traceless electronic remote control of different sets of biopharmaceuticals.

HEAT offers key advantages over previously reported temperature‐responsive sensors: it is activated by simple topical warming at a temperature that is perceived as pleasant by humans, and coupling the treatment with a positive sensation could improve patient compliance; it can precisely sense and discriminate fever‐range temperatures between 37 and 40 °C with sub‐degree precision enabling the detection of physiological temperature changes; it quickly returns to baseline activity upon a reduction in temperature, whereas endogenous transcription factors (such as HSF1 or NFAT) show prolonged activity after stimulation. Previously reported high‐temperature sensors have required an excess of 40 °C for activation, and such temperatures represent a life‐threatening medical emergency associated with pleiotropic heat‐shock responses.^[^ ^13^, ^16^ ^]^ Since hypothalamus‐coordinated thermostatic activities act to restore a normal core body temperature, which would be outside the sensors’ sensitivity and permissive temperature range,^[^ ^3^, ^61^ ^]^ these sensors are operational only transiently if at all in vivo. Emerging technologies such as short‐duration focused IR‐light‐induced temperature excursions following the incorporation of gold nanorods^[^ ^62^, ^63^ ^]^ could increase the efficiency of thermal stimulation and benefit the clinical translatability of both heat‐shock promoters and temperature‐sensing transcription factors.

Owing to its reversibility, HEAT may well be suited to monitor relapsing fever episodes in real‐time and could possibly be set up for closed‐loop therapeutic interventions. Considering its sub‐degree sensitivity, HEAT may in principle also be further refined to sense and react to transient sub‐fever body temperature deviations that occur during the circadian rhythm^[^ ^64^ ^]^ as well as fertility cycles^[^ ^65^ ^]^ or changes related to sleep disturbances, caloric restriction,^[^ ^66^ ^]^ alcohol consumption,^[^ ^67^ ^]^ strenuous exercise^[^ ^68^ ^]^ or a wide range of psychological factors.^[^ ^69^ ^]^ Wearables that continuously record body temperatures in real‐time have recently come into the limelight as a replacement for classic thermometry at random time points in order to monitor immunologic responses to viral infections and to track the spread of SARS‐CoV‐2.^[^ ^70^ ^]^ An improvement in sensitivity could further benefit applications deep in the body, where heat delivery is more efficiently counteracted by the body's homeostatic control of temperature. Genetically encoded protein thermometers such as HEAT that can directly, precisely and dynamically interface warm sensation or pyrexia with in‐situ actuation of biopharmaceutical production could provide the missing link between medical conditions associated with elevated temperature and preventive, chronic and acute interventions using cell‐based personalized medicine.

## 4 Experimental Section

### Plasmid Construction

Mammalian gene expression plasmids were constructed by standard molecular cloning techniques using restriction endonucleases (New England Biolabs, Ipswich, Massachusetts, USA; high‐fidelity enzymes were used whenever possible), followed by ligation with T4 DNA ligase (New England Biolabs, cat. no. M0202L). Before ligation the plasmid backbones were dephosphorylated with Antarctic phosphatase (New England Biolabs, cat. no. M0289L). PCR reactions were performed using Phusion High‐Fidelity DNA Polymerase (New England Biolabs, cat. no. M0530L) or Q5 High‐Fidelity DNA Polymerase (New England Biolabs, cat. no. M0491L). Details of all restriction enzymes and primers used for each plasmid are presented in Table S1 (Supporting Information). The plasmids were amplified in XL10 Gold chemically competent cells (New England Biolabs, cat. no. C2992) and DNA was extracted using a plasmid miniprep kit (Zymo Research, Irvine, California, USA, cat. no. D4054) or, for larger‐scale preparation, a ZymoPURE II Plasmid Midiprep Kit (Zymo Research, cat. no. D4200). Operator binding sites in reporter plasmids were introduced using custom oligonucleotides (Sigma Aldrich, Haverhill, UK) designed as complementary forward and reverse strands, which, upon annealing, form double‐stranded fragments with overhangs suitable for restriction endonuclease‐based cloning. For this, 10 × 10^−6^ m oligonucleotide mixture (5 × 10^−6^ m of each strand) was phosphorylated with T4 polynucleotide kinase (New England Biolabs, cat. no. M0201S) in T4 DNA ligase buffer (New England Biolabs, cat. no. M0202L) at 37 °C for 30 min. The mix was subsequently heated to 95 °C and slowly cooled to room temperature over 15 min. After 1:500 dilution, the double‐stranded phosphorylated fragment was used for cloning.

### Cell Culture

Human embryonic kidney cells (HEK‐293T, ATCC: CRL‐11268) were cultivated in Dulbecco's modified Eagle's medium (DMEM) (Thermo Fischer Scientific, Waltham, Massachusetts, USA, cat. no. 10566016) supplemented with 10% fetal bovine serum (FBS; Sigma‐Aldrich, Saint Louis, USA, cat. no. F7524, lot no. 022M3395) and penicillin (100 U)‐streptomycin (100 µg) solution (Sigma‐Aldrich, cat. no. P433) at 37 °C under a humidified atmosphere containing 5% CO_2_. Passaging of pre‐confluent HEK‐293 cultures was done by detaching cells through incubation in 0.05% trypsin‐EDTA (Life Technologies, Carlsbad, California, USA; cat. no. 25300‐054) for 5 min at 37 °C. Cells were transferred to 10 mL cell culture medium and centrifuged for 1 min at 200 x g, then the supernatant was discarded and the cells were resuspended in fresh medium at a standard cell density (1.5×10^5^ cells/mL), before reseeding them in cell culture plates. Cell number and viability were quantified using an electric field multichannel cell counting device (Casy Cell Counter and Analyzer Model TT, Roche Diagnostics GmbH, Rotkreuz, Switzerland). HeLa cells (HeLa, ATCC: CCL‐2), bone‐marrow‐derived immortalized mesenchymal stem/stromal cells (hMSC‐TERT) cells^[^ ^71^ ^]^ Hep G2 cells (HEPG2, ATCC: HB‐8065), baby hamster kidney cells (BHK‐21, ATCC: CCL‐10), HT‐1080 cells (HT‐1080, ATCC: CCL‐121), Hana3a (Hana3A, RRID:CVCL‐RW32), C2C12 cells (C2C12, ATCC: CRL‐1772), and Caco‐2 cells (Caco2, ATCC: HTB‐37) were cultivated as described for HEK‐293. INS‐1E cells (RRID: CVCL‐0351) were cultivated in RPMI‐1640 medium supplemented with 10% (v/v) FBS. Chinese hamster ovary cells (CHO‐K1, ATCC: CCL‐61) were cultivated in Ham's F‐12K (Kaighn's) medium (Thermo Fischer Scientific, Gibco F‐12K, cat. no. 21127‐022,) supplemented with 10% (v/v) FBS and 1% (v/v) streptomycin/penicillin. Fibroblast‐derived XM001 iPSCs^[^ ^72^ ^]^ were cultivated as described in the original reports. In brief, cells were cultured using mTeSR medium supplemented with normocin (50 µg mL^−1^) (Stem Cell Technologies, Vancouver, Canada, cat. no. 85850) on Geltrex‐coated cell culture plates in a 12‐well format (Invitrogen, cat. no. A1413202). Cells were routinely passaged as aggregates using ReLeSR (Stem Cell Technologies, cat. no. 05872) for cell detachment and re‐seeded at 25–35% confluence. For transfection experiments, cells were detached with Accutase (Life Technologies, cat. no. A1110501) and seeded in 24‐well plates in medium supplemented with 10 µM Y27632 (72302, Stem Cell Technologies).

### Transient Transfection

For plasmid transfection in a 96‐well format, cells were seeded at a density of 50000 cells per 1 cm^2^ in 100 µL medium and cultivated for 24 h. Then 50 µL serum and antibiotics‐free minimum essential medium MEM (Thermo Fischer Scientific, cat. no. 11095080) containing a 1:4 DNA:PEI mixture (polyethylenimine, MW 40000; Polysciences Inc., Warrington, USA, cat. no. 24765) with a total DNA amount of 350 ng cm^−2^ were added dropwise to the cells. After 8 h the culture medium was exchanged and the cells were cultivated for 24–72 h under specified treatment conditions before analysis. For other cell culture plate formats, the protocol was adjusted as necessary to take account of the area of the plates. Transfection of iPSCs was done using Lipofectamine Stem (Thermo Fischer Scientific, cat. no. STEM00015) and transfection of INS‐1E, C2C12 was done using Lipofectamine 3000 (Thermo Fischer Scientific, cat. no. L3000001), according to the manufacturer's protocols.

### Electronic Heating Patch Design

Small heating patches were constructed by fixing six resistors (Distrelec, Naenikon, Switzerland, cat. no. 300‐56‐888) connected in parallel (total resistance: 650 Ω) onto an insulated metal substrate. The substrate, which provides excellent heat dissipation, consisted of a 1 mm aluminum base plate, a 0.1 mm mid‐layer made of a glass‐epoxy resin‐ceramic compound for optimal heat conduction, and a 35 µm top copper layer that was in contact with the skin (Bungard Cotherm, Bungard Elektronik GmbH, Windeck, Germany). A thermistor (Heraeus Sensor Technology GmbH, Kleinostheim, Germany, cat. no. 17669005) was fixed to the aluminum base plate to monitor the temperature in real‐time. The heating patch was insulated using a two‐component heat‐conducting adhesive with thermal conductivity 0.84 W mK^−1^ (WLK30, Fischer Elektronik GmbH, Luedenscheid, Germany, cat. no. 18086141) and had a total area of 1 cm^2^. The system temperature was regulated by means of a JUMO cTRON compact controller (JUMO GmbH, Fulda, Germany) operated with a self‐optimizing proportional‐integral (PI) algorithm.

### Temperature Treatment of Engineered Cells

Heat‐inducible transgene expression in cultured human cells was achieved by placing parafilm‐sealed multi‐well cell culture dishes on a preheated Thermomixer Comfort heating block (Eppendorf AG, Hamburg, Germany) set at the required temperature. Long‐term temperature treatment was done by cultivation of the cells in an incubator heated to the required temperature. In reversibility experiments, SEAP concentrations were profiled 3 h after returning the cells to 37 °C to account for temperature transfer in the culture plates and accumulated mRNA.

### SEAP Quantification

Human placental secreted alkaline phosphatase (SEAP) concentration in the cell culture medium was determined by means of light‐absorbance time‐course measurement.^[^ ^73^ ^]^ For this, 20 µL of culture supernatant was mixed with 80 µL of ddH_2_O and heat‐inactivated for 30 min at 65 °C. Then, 80 µL of 2x SEAP buffer (20 × 10^−3^ m homoarginine, 1 × 10^−3^ m MgCl_2_, 21% (v/v) diethanolamine, pH 9.8) and 20 µL of 120 × 10^−3^ m para‐nitrophenyl phosphate (Fisher Scientific Acros Organics, Geel, Belgium, cat. no. 128860100) solution in 2x SEAP buffer were added to each well and the absorbance at 405 nm was measured at 37 °C using a Tecan M1000 plate reader (Tecan Group Ltd., Maennedorf, Switzerland). SEAP concentrations in serum were quantified using a chemiluminescence‐based assay (Roche Diagnostics, cat. no. 11779842001). In brief, 50 µL of heat‐inactivated serum (30 min, 65 °C), was centrifuged for 30 s at 5000x g and transferred to a well of a 96‐well plate containing 50 mL of inactivation buffer. The plate was incubated for 10 min at room temperature (21 °C), then 50 µL of substrate reagent was added to each well, and incubation was continued at r.t. for 10 min. Luminescence was measured using a Tecan M1000 plate reader and concentrations were calculated from a standard curve.

### NanoLuciferase Quantification

The concentration of NanoLuc in cell culture supernatants was measured by using the Nano‐Glo Luciferase Assay System (Promega, Madison, Wisconsin, USA, cat. no. N1110). In brief, 7.5 µL from each sample was mixed with 7.5 µL buffer/substrate mix (50:1) in 384 well plates (Greiner Bio One, Kremsmünster, Austria, cat. no 781076,) and incubated at room temperature (21 °C) for 10 min. Luminescence was measured with a Tecan M1000 plate reader (Tecan Group Ltd., Switzerland).

### Insulin Quantification

Recombinant mINS levels in culture supernatants of engineered cells were quantified using a mouse insulin ELISA kit according to the manufacturer's instructions (Mercordia, Uppsala, Sweden; cat. no. 10‐1247‐01). Optical density was measured at 450 nm on a Tecan M1000 plate reader and the corresponding concentrations were calculated in Prism 7 (GraphPad Software Inc., San Diego, California, USA) using a cubic‐spline regression based on the measured absorbances of manufacturer‐provided standard solutions.

### Generation of Monoclonal Stable Cell Lines

Polyclonal cell lines were generated by co‐transfection of a hyperactive Sleeping Beauty transposase (SB100x) expression vector in a 1:20 (weight/weight) ratio with the intended integration vectors containing SB recognition sites and encoding both a resistance marker and a fluorophore. The medium was exchanged 12 h after transfection and cells were incubated for 48 h before the addition of selection medium containing antibiotics corresponding to the resistance encoded by the integrated vectors. The following antibiotic concentrations were used during selection: zeocin 100 µg mL^−1^, puromycin 3 µg mL^−1^, blasticidin 8 µg mL^−1^. The cells were maintained in selection medium for three passages and then those with the highest expression levels were selected by means of fluorescence‐activated cell sorting (FACS) according to the signal intensity of the encoded fluorophores. Monoclonal cell populations were obtained by restrictive dilution at a ratio of 0.3 cells per well in a 96‐well format, followed by 2 weeks expansion before functional analysis.

### Animal Experiments

Temperature‐sensitive designer‐cell implants were produced by encapsulating transgenic HEK‐293 cells into coherent alginate‐poly‐(l‐lysine) beads (Na‐alginate, Buechi Labortechnik AG, Flawil, Switzerland; cat. no. 11061528; PLL, Sigma‐Aldrich, Saint Louis, USA, cat. no. L8662‐25G) using an Inotech Encapsulator Research Unit IE‐50R (Buechi Labortechnik AG, Flawil, Switzerland) with the following parameters: 200 µm nozzle, vibration frequency 1000 Hz, 900 V for bead dispersion, 20 mL syringe operated at a flow rate of 400 units. 500 µL of serum‐free DMEM containing 5×10^6^ microencapsulated transgenic cells was subcutaneously injected on the back of mice (Janvier Labs, Le Genest‐Saint‐Isle, France). Treated animals were kept for 24 h before experiments were conducted. Low‐grade persistent hyperthermia was used to simulate fever with a body temperature of 38 °C according to an established protocol.^[^ ^43^, ^44^ ^]^ In brief, animals were given a 500 µL i.p. injection of physiological saline, and kept in cages in a ventilated incubator set to 36 °C. Access to food and water was provided ad libitum. To ensure that dehydration did not occur, mice received oral gavage of 200 µL water twice daily. For simulation of high‐grade fever, immobilized mice were sequentially subjected to an environmental temperature of 39.5 °C for 2 h followed by a 15 min resting time at room temperature (21 °C) for a total of 9 h d^−1^ as described in an established protocol.^[^ ^45^ ^]^ Before the initial stimulation, mice received i.p. injections of 500 µL physiological saline and oral gavage of 200 µL during the subsequent rest phases. Skin temperature was recorded at the implant site with a Uni‐T UT305H infrared thermometer, which provides an accurate estimate of the body's core temperature.^[^ ^74^ ^]^ For extracorporeal transcutaneous temperature control of designer cell implants in mice, the custom‐designed heating patch was fixed on the shaved backs of mice and adjusted to two degrees higher temperature than the desired temperature in the implants, to account for temperature dissipation through the skin. Type‐1 diabetes was induced in mice as previously described.^[^ ^57^ ^]^ Mice were fasted for 12 h overnight and injected with 60 mg kg^−1^ of streptozotocin (Sigma‐Aldrich, cat. no. S0130) for 5 d. Fasting blood glucose was measured after 8 h of food restriction using a clinically licensed glucometer (Contour Next, Bayer Healthcare, Germany). Postprandial glycemic excursions were simulated by i.p. injection of 1.5 g kg^−1^ glucose, and blood glucose was profiled every 15 to 30 min for the following 2 h. For analysis of SEAP levels, blood serum was collected using Microtainer serum‐separating tubes according to the manufacturer's protocol (Becton Dickinson, cat. no. 365967) and quantified as described above. All experiments involving animals were performed according to the directive of the European Community Council (2010/63/EU), approved by the French Republic (project no. DR2018‐40v5, APAFIS #16753), and carried out by Ghislaine Charpin‐El Hamri (license no. 69266309) at the Institut Universitaire de Technologie of the Université Claude Bernard Lyon 1, F‐696226, Villeurbanne Cedex, France.

### Statistical Analysis

Details of data presentation, sample size (*n*), statistical analysis and significance of differences are given in the figure captions. Statistical evaluation was conducted by using an unpaired Student's two‐tailed *t*‐test for comparing two sets of data and one‐way ANOVA for multiple comparisons as implemented in Prism GraphPad 7 (GraphPad Software Inc., San Diego, California, USA).

## Conflict of Interest

The authors declare no conflict of interest.

## Author Contributions

B.A.S. and M.F. conceived the project and designed the experiments. B.A.S, A.P.T., M.M., A.B., G.C., S.X., and K.K. conducted the experiments. B.A.S., A.P.T., and M.F. analyzed the data and wrote the manuscript.

## Supporting information

Supporting Information

## Data Availability

Detailed vector information is provided in Table S1 (Supporting Information). All vectors and data are freely available upon request.

## References

[1] N. Merino , H. S. Aronson , D. P. Bojanova , J. Feyhl‐Buska , M. L. Wong , S. Zhang , D. Giovannelli , Front. Microbiol. 2019, 10, 780. 31037068 10.3389/fmicb.2019.00780 PMC6476344

[2] G. N. Somero , J. Exp. Biol. 2010, 213, 912. 20190116 10.1242/jeb.037473

[3] S. F. Morrison , K. Nakamura , Annu. Rev. Physiol. 2019, 81, 285. 30256726 10.1146/annurev-physiol-020518-114546

[4] C. L. Tan , E. K. Cooke , D. E. Leib , Y. C. Lin , G. E. Daly , C. A. Zimmerman , Z. A. Knight , Cell 2016, 167, 47.e15. 27616062 10.1016/j.cell.2016.08.028 PMC5062957

[5] R. Paricio‐Montesinos , F. Schwaller , A. Udhayachandran , F. Rau , J. Walcher , R. Evangelista , J. Vriens , T. Voets , J. F. A. Poulet , G. R. Lewin , Neuron 2020, 106, 830.e3. 32208171 10.1016/j.neuron.2020.02.035 PMC7272120

[6] C. Arrigoni , D. L. Minor , Pflugers Arch. 2018, 470, 733. 29340775 10.1007/s00424-017-2102-z PMC5945320

[7] K. Castillo , I. Diaz‐Franulic , J. Canan , F. Gonzalez‐Nilo , R. Latorre , Phys. Biol. 2018, 15, 021001. 29135465 10.1088/1478-3975/aa9a6f

[8] C. H. Tan , P. A. McNaughton , Nature 2016, 536, 460. 27533035 10.1038/nature19074 PMC5720344

[9] D. Ogoina , J. Infect. Public Health 2011, 4, 108. 21843857 10.1016/j.jiph.2011.05.002

[10] D. A. Milner , Cold Spring Harbor Perspect. Med. 2018, 8, a025569. 10.1101/cshperspect.a025569 PMC5749143 28533315

[11] B. Mizrahi , S. Shilo , H. Rossman , N. Kalkstein , K. Marcus , Y. Barer , A. Keshet , N. Shamir‐Stein , V. Shalev , A. E. Zohar , G. Chodick , E. Segal , Nat. Commun. 2020, 11, 6208. 33277494 10.1038/s41467-020-20053-y PMC7718370

[12] N. Prajitha , S. S. Athira , P. V. Mohanan , Immunol. Lett. 2018, 204, 38. 30336182 10.1016/j.imlet.2018.10.006

[13] P. Kiekkas , D. Aretha , N. Bakalis , I. Karpouhtsi , C. Marneras , G. I. Baltopoulos , Aust. Crit. Care. 2013, 26, 130. 23199670 10.1016/j.aucc.2012.10.004

[14] E. J. Noonan , R. F. Place , C. Giardina , L. E. Hightower , Cell Stress Chaperones 2007, 12, 393. 18229458 10.1379/CSC-278e.1 PMC2134801

[15] H. Saibil , Nat. Rev. Mol. Cell Biol. 2013, 14, 630. 24026055 10.1038/nrm3658 PMC4340576

[16] D. B. Mahat , H. H. Salamanca , F. M. Duarte , C. G. Danko , J. T. Lis , Mol. Cell 2016, 62, 63. 27052732 10.1016/j.molcel.2016.02.025 PMC4826300

[17] A. D. Al‐Jawdah , I. G. Ivanova , H. Waller , N. D. Perkins , J. H. Lakey , D. T. Peters , BMC Microbiol. 2019, 19, 68. 30922226 10.1186/s12866-019-1444-4 PMC6440114

[18] R. Hurme , K. D. Berndt , S. J. Normark , M. Rhen , Cell 1997, 90, 55. 9230302 10.1016/s0092-8674(00)80313-x

[19] F. Righetti , F. Narberhaus , Front. Cell. Infect. Microbiol. 2014, 4, 132. 25279353 10.3389/fcimb.2014.00132 PMC4166951

[20] M. Falconi , B. Colonna , G. Prosseda , G. Micheli , C. O. Gualerzi , EMBO J. 1998, 17, 7033. 9843508 10.1093/emboj/17.23.7033 PMC1171051

[21] P. Mandin , J. Johansson , Mol. Microbiol. 2020, 113, 588. 31971637 10.1111/mmi.14468

[22] R. R. Naik , S. M. Kirkpatrick , M. O. Stone , Biosens. Bioelectron. 2001, 16, 1051. 11679288 10.1016/s0956-5663(01)00226-3

[23] S. Kiyonaka , T. Kajimoto , R. Sakaguchi , D. Shinmi , M. Omatsu‐Kanbe , H. Matsuura , H. Imamura , T. Yoshizaki , I. Hamachi , T. Morii , Y. Mori , Nat. Methods 2013, 10, 1232. 24122038 10.1038/nmeth.2690

[24] D. I. Piraner , M. H. Abedi , B. A. Moser , A. Lee‐Gosselin , M. G. Shapiro , Nat. Chem. Biol. 2017, 13, 75. 27842069 10.1038/nchembio.2233

[25] D. I. Piraner , Y. Wu , M. G. Shapiro , ACS Synth. Biol. 2019, 8, 2256. 31491082 10.1021/acssynbio.9b00275

[26] M. Boorsma , L. Nieba , D. Koller , M. F. Bachmann , J. E. Bailey , W. A. Renner , Nat. Biotechnol. 2000, 18, 429. 10748525 10.1038/74493

[27] W. Weber , R. R. Marty , N. Link , M. Ehrbar , B. Keller , C. C. Weber , A. H. Zisch , C. Heinzen , V. Djonov , M. Fussenegger , Nucleic Acids Res. 2003, 31, 69e. 10.1093/nar/gng069 PMC162344 12799458

[28] P. Bai , Y. Liu , S. Xue , G. C. El Hamri , P. Saxena , H. Ye , M. Xie , M. Fussenegger , Nat. Med. 2019, 25, 1266. 31285633 10.1038/s41591-019-0501-8

[29] M. H. Abedi , J. Lee , D. I. Piraner , M. G. Shapiro , ACS Synth. Biol. 2020, 9, 1941. 32786924 10.1021/acssynbio.0c00238

[30] D. E. Kruse , M. A. Mackanos , C. E. O'Connell‐Rodwell , C. H. Contag , K. W. Ferrara , Phys. Med. Biol. 2008, 53, 3641. 18562783 10.1088/0031-9155/53/13/017 PMC2763418

[31] M. Yamaguchi , A. Ito , A. Ono , Y. Kawabe , M. Kamihira , ACS Synth. Biol. 2014, 3, 273. 24144205 10.1021/sb4000838

[32] P. S. Yarmolenko , E. J. Moon , C. Landon , A. Manzoor , D. W. Hochman , B. L. Viglianti , M. W. Dewhirst , Int. J. Hyperthermia 2011, 27, 320. 21591897 10.3109/02656736.2010.534527 PMC3609720

[33] H. Arami , A. Khandhar , D. Liggitt , K. M. Krishnan , Chem. Soc. Rev. 2015, 44, 8576. 26390044 10.1039/c5cs00541h PMC4648695

[34] J. R. Deuis , I. Vetter , Temperature 2016, 3, 199. 10.1080/23328940.2016.1157668 PMC4965000 27857950

[35] V. Ortner , A. Ludwig , E. Riegel , S. Dunzinger , T. Czerny , Cell Stress Chaperones 2015, 20, 277. 25168173 10.1007/s12192-014-0540-5 PMC4326385

[36] T. Haltenhof , A. Kotte , F. De Bortoli , S. Schiefer , S. Meinke , A. K. Emmerichs , K. K. Petermann , B. Timmermann , P. Imhof , A. Franz , B. Loll , M. C. Wahl , M. Preußner , F. Heyd , Mol. Cell 2020, 78, 57.e4. 32059760 10.1016/j.molcel.2020.01.028

[37] M. Preußner , G. Goldammer , A. Neumann , T. Haltenhof , P. Rautenstrauch , M. Müller‐McNicoll , F. Heyd , Mol. Cell 2017, 67, 433.e4. 28689656 10.1016/j.molcel.2017.06.006

[38] P. Saxena , B. C. Heng , P. Bai , M. Folcher , H. Zulewski , M. Fussenegger , Nat. Commun. 2016, 7, 11247. 27063289 10.1038/ncomms11247 PMC4831023

[39] F. Sedlmayer , D. Aubel , M. Fussenegger , Nat. Biomed. Eng. 2018, 2, 399. 31011195 10.1038/s41551-018-0215-0

[40] H. Ye , M. D. El Baba , R. W. Peng , M. Fussenegger , Science 2011, 332, 1565. 21700876 10.1126/science.1203535

[41] M. Kurokawa , M. Imakita , C. A. Kumeda , K. Shiraki , J. Med. Virol. 1996, 50, 152. 8915881 10.1002/(SICI)1096-9071(199610)50:2<152::AID-JMV8>3.0.CO;2-9

[42] Q. Jiang , A. S. Cross , I. S. Singh , T. T. Chen , R. M. Viscardi , J. D. Hasday , Infect. Immun. 2000, 68, 1265. 10678936 10.1128/iai.68.3.1265-1270.2000 PMC97277

[43] H. Sareh , M. E. Tulapurkar , N. G. Shah , I. S. Singh , J. D. Hasday , Cell Stress Chaperones 2011, 16, 297. 21080137 10.1007/s12192-010-0240-8 PMC3077225

[44] A. B. Lipke , G. Matute‐Bello , R. Herrero , K. Kurahashi , V. A. Wong , S. M. Mongovin , T. R. Martin , J. Immunol. 2010, 184, 3801. 20200273 10.4049/jimmunol.0903191 PMC2865890

[45] V. Duhan , N. Joshi , P. Nagarajan , P. Upadhyay , J. Visualized Exp. 2012, e3801. 10.3791/3801 PMC3486748 22951580

[46] E. T. Rolls , F. Grabenhorst , B. A. Parris , Neuroimage 2008, 41, 1504. 18468458 10.1016/j.neuroimage.2008.03.005

[47] B. P. Kramer , A. U. Viretta , M. D. El Baba , D. Aubel , W. Weber , M. Fussenegger , Nat. Biotechnol. 2004, 22, 867. 15184906 10.1038/nbt980

[48] K. Rössger , G. Charpin‐El‐Hamri , M. Fussenegger , Nat. Commun. 2013, 4, 2825. 24281397 10.1038/ncomms3825 PMC3868331

[49] L. Scheller , T. Strittmatter , D. Fuchs , D. Bojar , M. Fussenegger , Nat. Chem. Biol. 2018, 14, 723. 29686358 10.1038/s41589-018-0046-z

[50] M. Gossen , S. Freundlieb , G. Bender , G. Müller , W. Hillen , H. Bujard , Science 1995, 268, 1766. 7792603 10.1126/science.7792603

[51] W. Weber , W. Bacchus , M. Daoud‐El Baba , M. Fussenegger , Nucleic Acids Res. 2007, 35, e116. 17827215 10.1093/nar/gkm466 PMC2034481

[52] M. Xie , H. Ye , G. Charpin‐El Hamri , M. Fussenegger , Nucleic Acids Res. 2014, 42, e116. 25030908 10.1093/nar/gku545 PMC4132709

[53] R. Kojima , D. Aubel , M. Fussenegger , Cell. Mol. Life Sci. 2020, 77, 3567. 32185403 10.1007/s00018-020-03486-y PMC7452942

[54] V. Ortner , C. Kaspar , C. Halter , L. Töllner , O. Mykhaylyk , J. Walzer , W. H. Günzburg , J. A. Dangerfield , C. Hohenadl , T. Czerny , J. Controlled Release 2012, 158, 424. 10.1016/j.jconrel.2011.12.006 PMC3329627 22197778

[55] S. A. Stanley , L. Kelly , K. N. Latcha , S. F. Schmidt , X. Yu , A. R. Nectow , J. Sauer , J. P. Dyke , J. S. Dordick , J. M. Friedman , Nature 2016, 531, 647. 27007848 10.1038/nature17183 PMC4894494

[56] S. A. Stanley , J. E. Gagner , S. Damanpour , M. Yoshida , J. S. Dordick , J. M. Friedman , Science 2012, 336, 604. 22556257 10.1126/science.1216753 PMC3646550

[57] K. Krawczyk , S. Xue , P. Buchmann , G. Charpin‐El‐Hamri , P. Saxena , M. D. Hussherr , J. Shao , H. Ye , M. Xie , M. Fussenegger , Science 2020, 368, 993. 32467389 10.1126/science.aau7187

[58] A. Zaldumbide , S. Weening , S. J. Cramer , M. J. W. E. Rabelink , J. Verhaagen , R. C. Hoeben , Biotechnol. Lett. 2010, 32, 749. 20155386 10.1007/s10529-010-0218-8

[59] P. Saeedi , I. Petersohn , P. Salpea , B. Malanda , S. Karuranga , N. Unwin , S. Colagiuri , L. Guariguata , A. A. Motala , K. Ogurtsova , J. E. Shaw , D. Bright , R. Williams , Diabetes Res. Clin. Pract. 2019, 157, 107843. 31518657 10.1016/j.diabres.2019.107843

[60] M. Baghelani , Z. Abbasi , M. Daneshmand , P. E. Light , Sci. Rep. 2020, 10, 12980. 32737348 10.1038/s41598-020-69547-1 PMC7395170

[61] C. L. Tan , Z. A. Knight , Neuron 2018, 98, 31. 29621489 10.1016/j.neuron.2018.02.022 PMC6034117

[62] I. C. Miller , M. Gamboa , J. Castro , J. P. Maenza , G. A. Weis , Kwong , ACS Synth. Biol. 2018, 7, 1167. 29579381 10.1021/acssynbio.7b00455 PMC5929470

[63] L. Gamboa , E. V. Phung , H. Li , J. P. Meyers , A. C. Hart , I. C. Miller , G. A. Kwong , ACS Chem. Biol. 2020, 15, 533. 31904924 10.1021/acschembio.9b01005 PMC7035993

[64] R. Lok , M. J. van Koningsveld , M. C. M. Gordijn , D. G. M. Beersma , R. A. Hut , J. Pineal Res. 2019, 67, e12583. 31033013 10.1111/jpi.12583 PMC6767594

[65] M. Shilaih , B. M. Goodale , L. Falco , F. Kübler , V. De Clerck , B. Leeners , Biosci. Rep. 2018, 38, 10.1042/BSR20171279. PMC6265623 29175999

[66] J. R. Speakman , S. E. Mitchell , Mol. Aspects Med. 2011, 32, 159. 21840335 10.1016/j.mam.2011.07.001

[67] L. I. Crawshaw , H. Wallace , J. Crabbe , Clin. Exp. Pharmacol. Physiol. 1998, 25, 150. 9493506 10.1111/j.1440-1681.1998.tb02195.x

[68] P. J. O'Connor , M. J. Breus , S. D. Youngstedt , Physiol. Behav. 1998, 64, 213. 9748085 10.1016/s0031-9384(98)00049-3

[69] K. P. Wright , J. T. Hull , C. A. Czeisler , Am. J. Physiol.: Regul., Integr. Comp. Physiol. 2002, 283, R1370. 12388468 10.1152/ajpregu.00205.2002

[70] B. L. Smarr , K. Aschbacher , S. M. Fisher , A. Chowdhary , S. Dilchert , K. Puldon , A. Rao , F. M. Hecht , A. E. Mason , Sci. Rep. 2020, 10, 21640. 33318528 10.1038/s41598-020-78355-6 PMC7736301

[71] J. L. Simonsen , C. Rosada , N. Serakinci , J. Justesen , K. Stenderup , S. I. S. Rattan , T. G. Jensen , M. Kassem , Nat. Biotechnol. 2002, 20, 592. 12042863 10.1038/nbt0602-592

[72] X. Wang , M. Sterr , I. Burtscher , S. Chen , A. Hieronimus , F. Machicao , H. Staiger , H. U. Häring , G. Lederer , T. Meitinger , F. M. Cernilogar , G. Schotta , M. Irmler , J. Beckers , M. H. de Angelis , M. Ray , C. V. E. Wright , M. Bakhti , H. Lickert , Mol. Metab. 2018, 9, 57. 29396371 10.1016/j.molmet.2018.01.011 PMC5870105

[73] J. Berger , J. Hauber , R. Hauber , R. Geiger , B. R. Cullen , Gene 1988, 66, 1. 3417148 10.1016/0378-1119(88)90219-3

[74] J. Mei , N. Riedel , U. Grittner , M. Endres , S. Banneke , J. V. Emmrich , Sci. Rep. 2018, 8, 3526. 29476115 10.1038/s41598-018-22020-6 PMC5824949

