# Supplementary material for: Genetically Encoded Protein Thermometer Enables Precise Electrothermal Control of Transgene Expression
Source: Adv Sci (Weinh). 2021 Sep 8;8(21):2101813. doi: 10.1002/advs.202101813 (PMC8564464; doi:10.1002/advs.202101813)
Supplement: Supplementary file 1 — Supporting Information [file ADVS-8-2101813-s001.pdf]

© 2020 Wiley-VCH GmbH

ADVANCED SCIENCE
Open Access

Supporting Information

for *Adv. Sci.,* DOI: 10.1002/advs.202101813

Genetically Encoded Protein Thermometer Enables Precise Electrothermal Control of Transgene Expression

*Bozhidar-Adrian Stefanov, Ana P. Teixeira, Maysam Mansouri, Adrian Bertschi, Krzysztof Krawczyk, Ghislaine Charpin-El Hamri, Shuai Xue, Martin Fussenegger**

Supplementary Information

Genetically encoded protein thermometer enables precise electrothermal control of transgene expression

Bozhidar-Adrian Stefanov, Ana P. Teixeira, Maysam Mansouri, Adrian Bertschi, Krzysztof Krawczyk, Ghislaine Charpin-El Hamri, Shuai Xue, Martin Fussenegger*

*Correspondence: fussenegger@bsse.ethz.ch

Figures S1 to S10
Table S1
References

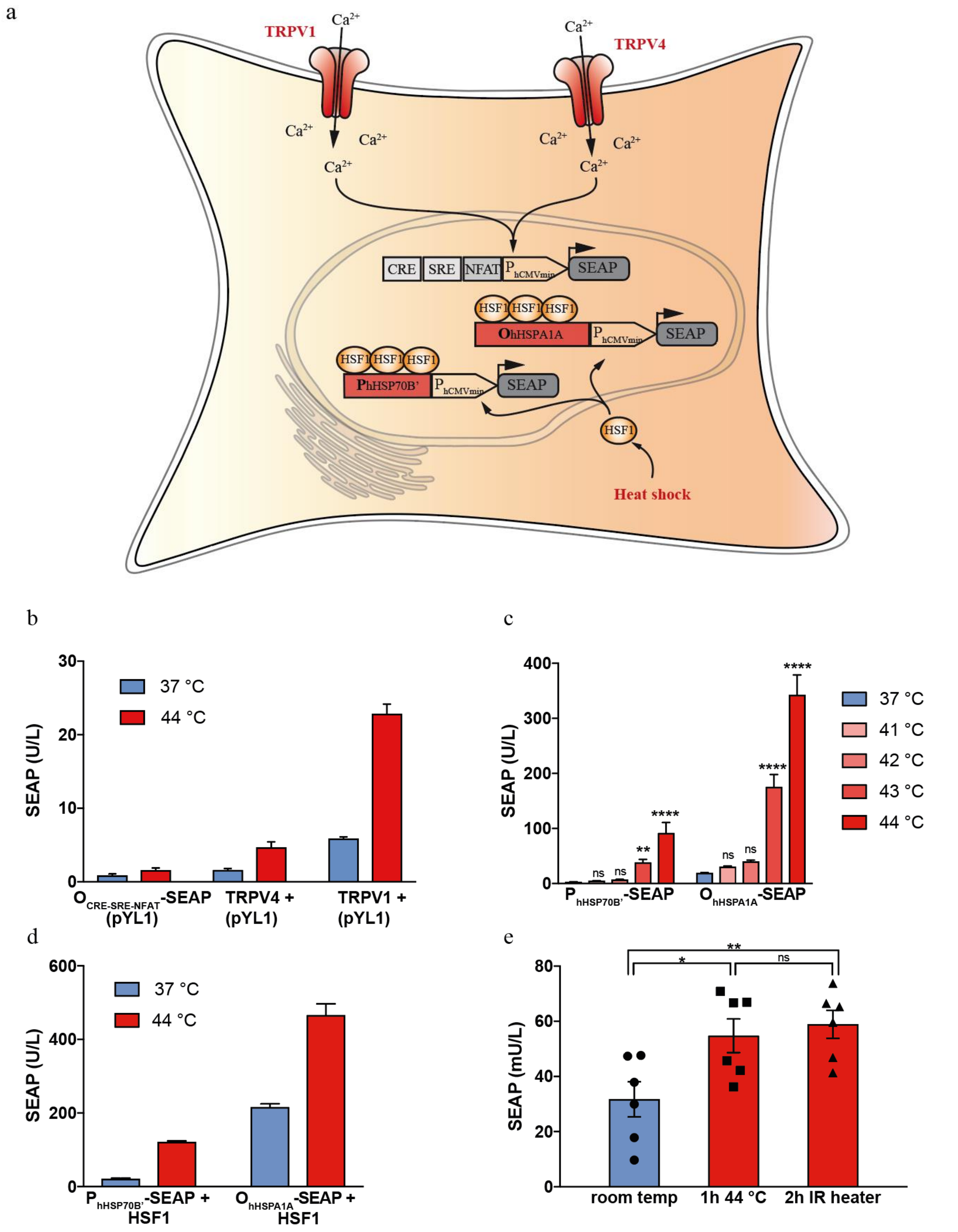

Supplementary Figure 1 | Rewiring mammalian temperature-sensing components for heat-inducible transgene expression. **a** Calcium-permeable temperature-sensitive ion channels can be rerouted to synthetic promoters activated by calcium signaling. Heat shock-activated HSF1 can induce gene expression from synthetic promoters with HSF1 recognition sequences. **b** HEK-293 cells transiently transfected with either rat TRPV4 or rat TRPV1 and a reporter

vector for SEAP gene expression controlled by a synthetic promoter containing CRE, SRE and NFAT binding sites (pYL1) were exposed to 44 °C for 2 h, and then the SEAP concentration in the medium was quantified. **c** HEK-293 cells transfected with vectors encoding SEAP expression under the control of either human HSP70B’ promoter or six synthetic heat shock elements (HSE) from the human HSPA1A promoter upstream of a minimal promoter were exposed for 2 h to different temperatures from 37°C to 44 °C and then incubated for 24 h at 37 °C before quantification of SEAP in the culture supernatant. **d** HSF1 co-expression increases non-induced baseline SEAP levels from HSPA1A-derived synthetic promoter. HEK-293 cells were transfected with HSP70B’ or HSPA1A based SEAP reporter vector and a constitutively expressed heat shock factor 1 (HSF1)-encoding vector. For induction, the cells were pulsed for 2 h at 44 °C before incubation for 24 h at 37 °C. The reporter gene product, SEAP, was quantified in the supernatant. **e** HEK-293 cells transgenic for SEAP reporter gene expression from the synthetic HSP70B’-derived promoter were alginate-encapsulated and subcutaneously implanted on the back of mice. The cells were stimulated either by a 44 °C pre-warmed heating pad for 1 h or by placing the mice under an infrared lamp for 2 hours. SEAP concentration was quantified in serum samples collected 24 h after stimulation. Data in **b**, **c**, **d** are shown as bar graphs of mean ± SD of n = 3 biologically independent samples, representative of three independent experiments. Data in **e** are shown as a bar graph of mean ± SEM of 6 mice (n = 6) Statistical analysis in **c**, **e** was done by one-way ANOVA: ns not significant, * $P < 0.05$, ** $P < 0.01$, *** $P < 0.001$, **** $P < 0.0001$.

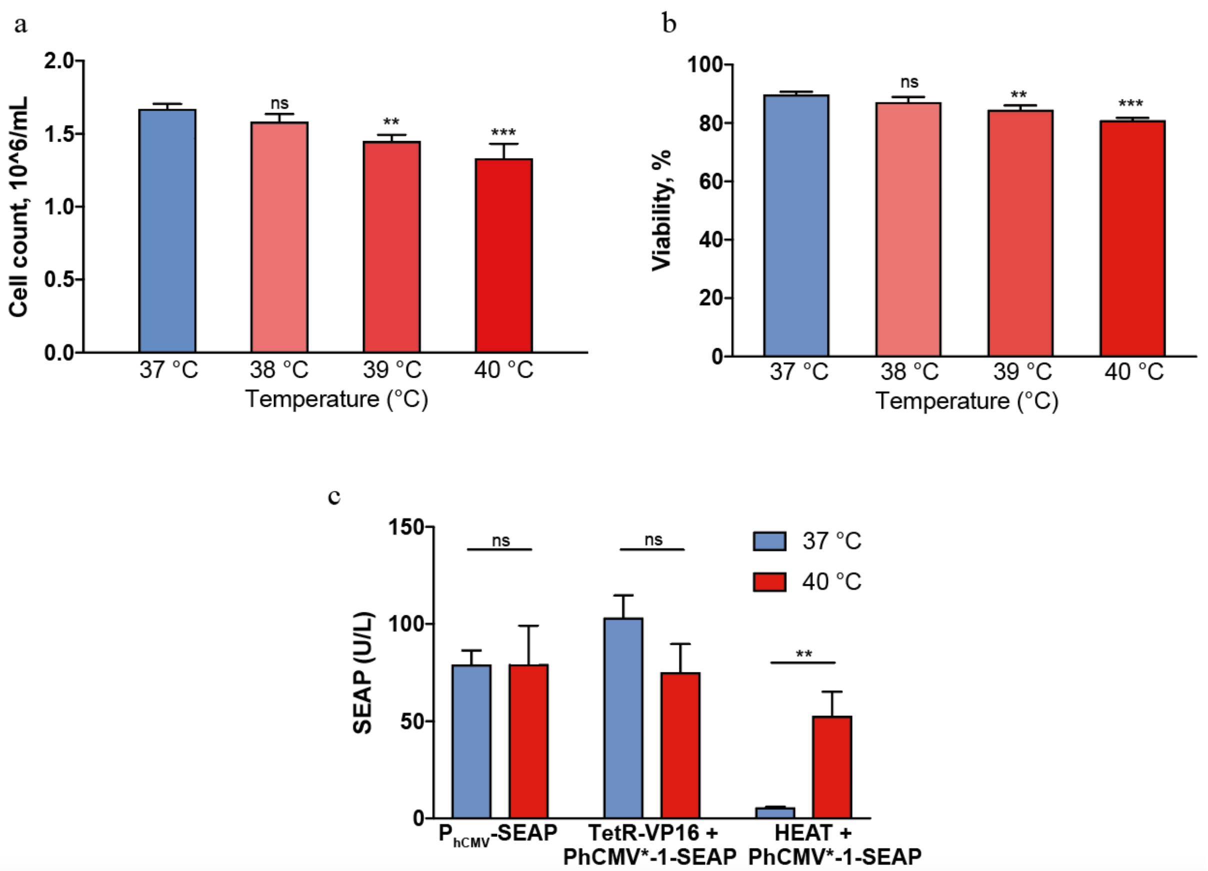

Supplementary Figure 2 | Effects of temperature on proliferation, viability and transgene expression of HEK-293 cells. **a** HEK-293 cell densities after a 72 h incubation period at 37, 38, 39 and 40 °C. The cells were seeded in 96-well plates at a density of 0.2 $\times 10^6$ cell/mL and harvested after 72 h for cell counting. **b** Viability of HEK-293 cells after a 72 h incubation period at 37, 38, 39 and 40 °C. The cells were seeded in 96-well plates at a density of 0.2 x10^6 cell/mL and harvested after 72 h. **c** SEAP reporter gene expression from a constitutive P_{hCMV} promoter, from the inducible tetracycline-controlled $P_{hCMV*-1}$ promoter in cells constitutively co-expressing the tetracycline transactivator tTA (TetR-VP16), and from the $P_{hCMV*-1}$ promoter were quantified in HEAT-transfected HEK-293 cells cultured for 24h at 37 °C and 40 °C. Data are shown as bar graphs of mean ± SD of n = 3 biologically independent samples, representative of three independent experiments. Multiple comparisons in **a**, **b** was done by one-way ANOVA comparing the means to the 37 °C control, and comparisons in **c** were done using a two-tailed Student's t-test: ns not significant, * $P < 0.05$, ** $P < 0.01$, *** $P < 0.001$.

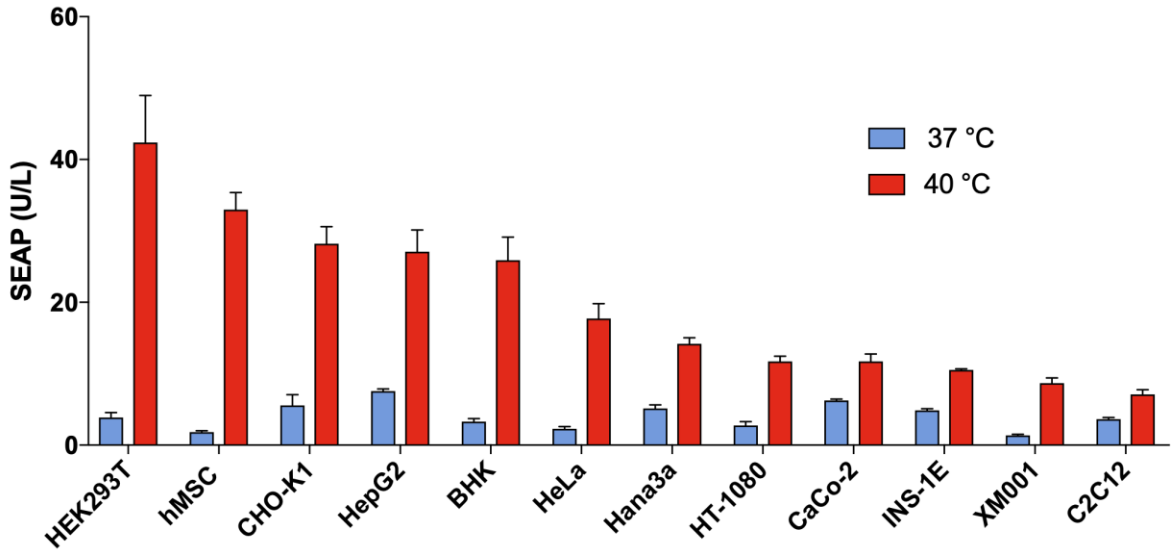

Supplementary Figure 3 | Exploring applicable fields through quantification of SEAP reporter gene expression in mammalian cells originated from diverse tissues and transiently transfected with HEAT (pBS707) and a $P_{hCMV*-1}$ controlled SEAP reporter vector, upon incubation at 37 and 40 °C. To provide a first non-comparative qualitative performance validation across different cell types, the cells were transfected using the same non-optimized generic transfection protocol and equal amounts of DNA. For higher performance, the transfection protocols need to be adapted for each cell type. Data are shown as a bar graph of mean ± SD of n = 3 biologically independent samples, representative of three independent experiments.

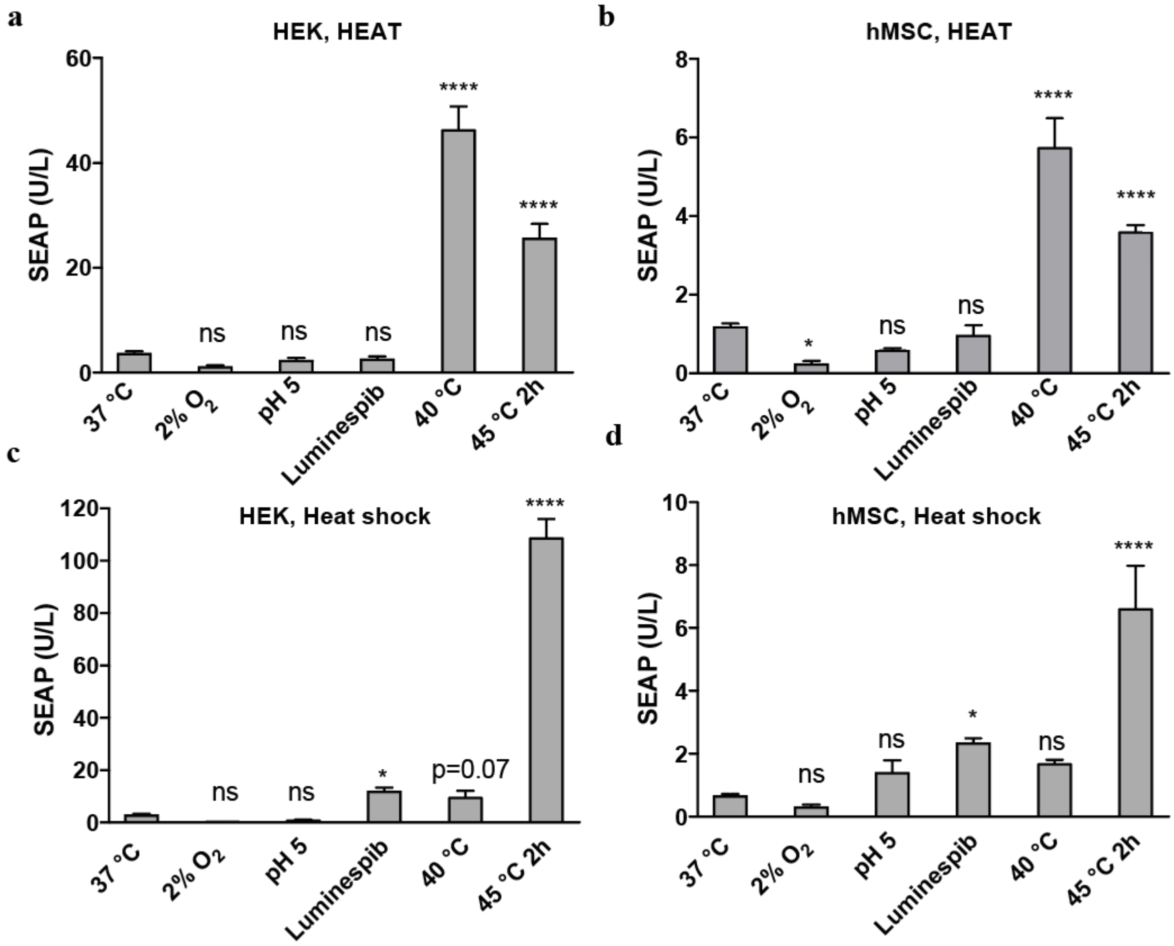

Supplementary Figure 4 | Testing the orthogonality of HEAT and the HSPA1A-derived synthetic heat shock promoter in HEK-293 cells and hMSCs. **a-d** SEAP levels were quantified in the culture supernatants of transiently transfected cells subjected to the indicated treatments for 24 hr. **a** HEK-293 cells and **b** hMSCs expressing HEAT and the corresponding $P_{hCMV1\text{-}*}$ controlled SEAP reporter gene. **c** HEK-293 cells and **d** hMSCs expressing SEAP reporter gene from the HSPA1A-derived synthetic heat shock promoter. Data are shown as bar graphs of mean ± SD of n = 3 biologically independent samples, representative of three independent experiments. Statistical analysis was done by one-way ANOVA compared to the 37°C control: ns not significant, * P < 0.05, ** P < 0.01, *** P < 0.001, **** P < 0.0001.

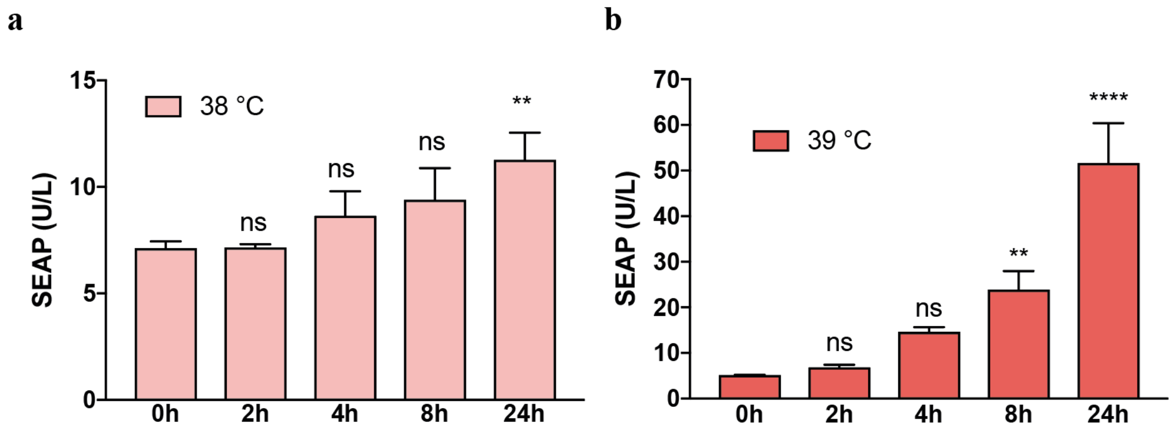

Supplementary Figure 5 | a-b Time-response relationship of HEAT (pBS707) for transcription from a $P_{hCMV*-1}$ controlled SEAP reporter gene expression vector in transfected HEK-293 cells incubated for the indicated duration at **a** 38 °C or **b** 39 °C and then returned to 37 °C for the rest of the 24 h period. Data are shown as bar graphs of mean ± SD of n = 3 biologically independent samples, representative of three independent experiments. Statistical analysis was done by one-way ANOVA compared to the 0 h control: ns not significant, * $P < 0.05$, ** $P < 0.01$, *** $P < 0.001$, **** $P < 0.0001$.

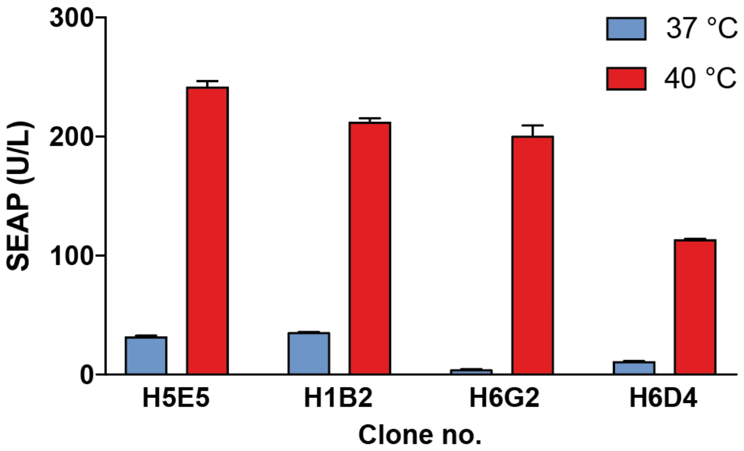

Supplementary Figure 6 | Selection of best-performing cell lines for heat-responsive secretion of SEAP reporter. Monoclonal populations derived from polyclonal cell lines stably expressing HEAT (TlpA$_{39}$-FFF-tTA) and SEAP from an inducible P$_{hCMV*-1}$ promoter were pre-screened for inducibility of the reporter gene at 40 °C versus 37 °C, and the best populations in terms of fold-change were split in triplicate onto two plates. After incubation for 72 h at the appropriate temperature, SEAP was quantified in samples from the supernatants. Clone H6G2 was selected as the FeverSense monoclonal cell line as it showed the best-in-class fold change. Data are shown as a bar graph of mean ± SD of n = 3 biologically independent samples, representative of three independent experiments.

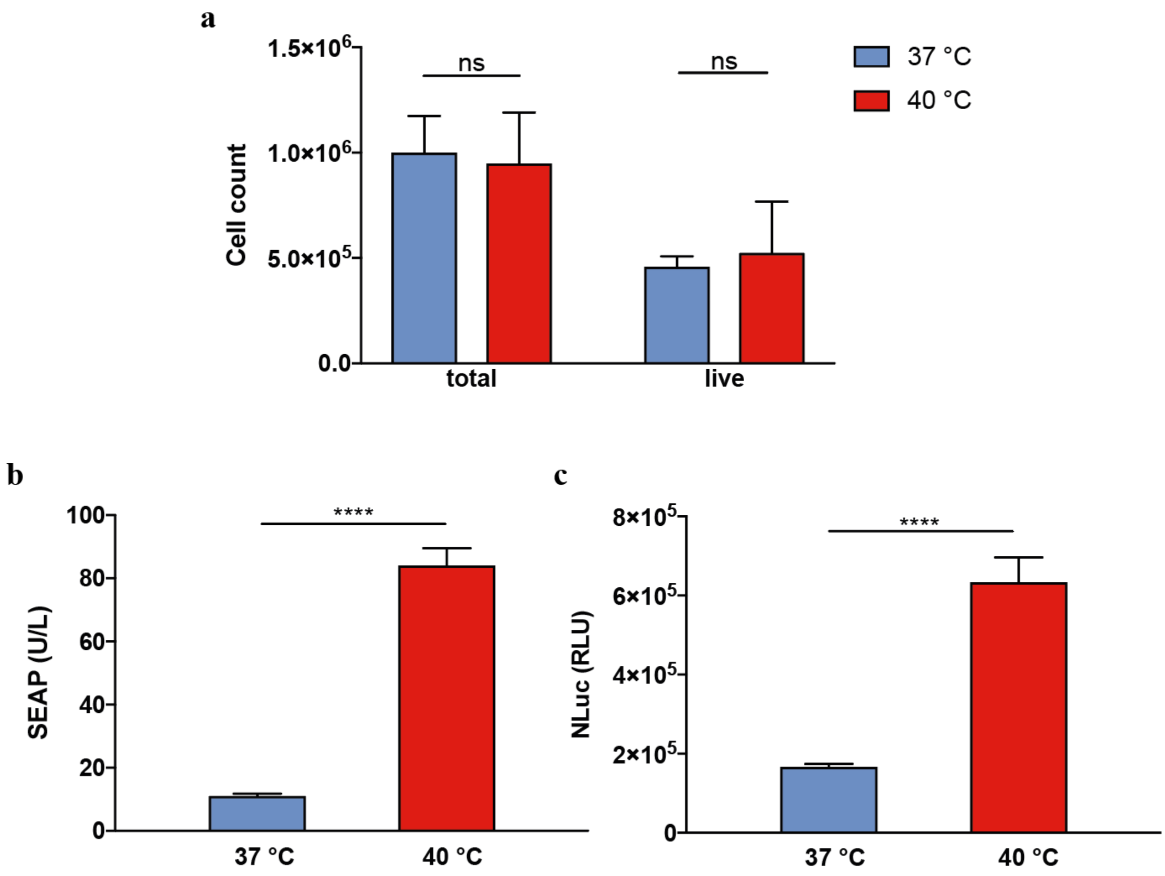

Supplementary Figure 7 | Functionality of HEAT in human mesenchymal stem cells (hMSCs). **a** Transiently transfected hMSCs were maintained for 24 h after transfection at the indicated temperatures, then the total cell count was determined, and a trypan blue stain was used to identify viable cells. hMSCs stably expressing HEAT and **b** SEAP or **c** nanoluciferase reporter gene controlled by the $P_{hCMV1-*}$ promoter were incubated at the indicated temperatures. Data are shown as bar graphs of mean ± SD of n = 3 biologically independent samples, representative of three independent experiments. Statistical analysis was done using a two-tailed Student’s t-test: ns not significant, **** P < 0.0001.

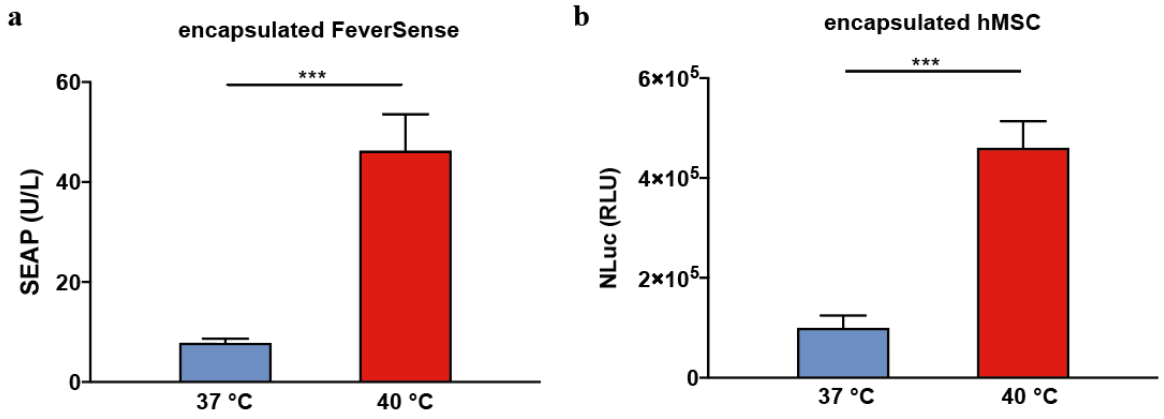

Supplementary Figure 8 | HEAT system functionality in alginate-encapsulated cells. **a** SEAP expression by alginate-encapsulated FeverSense cells maintained at the indicated temperatures. **b** NLuc expression by alginate-encapsulated hMSCs stably expressing HEAT and maintained at the indicated temperatures for 48 h. Data are shown as bar graphs of mean ± SD of n = 3 biologically independent samples, representative of three independent experiments. Statistical analysis was done using a two-tailed Student's t-test: ns not significant, *** P < 0.001.

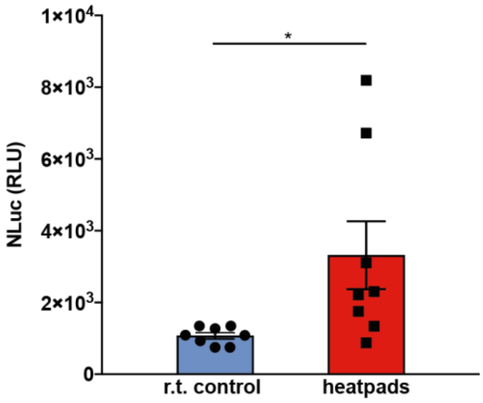

Supplementary Figure 9 | Nanoluciferase reporter gene levels in the serum of mice with subcutaneously implanted hMSCs stably expressing HEAT and a $P_{hCMV*-1}$ controlled reporter vector 24 h after implantation and at the start of the experiment. The treatment was applied by an external heating source fixed onto the back of the mice above the encapsulated cells. Data are shown as a bar graph of mean ± SEM with overlayed individual values of 8 mice (n = 8). Statistical analysis was done using a two-tailed Student's t-test: ns not significant, * $P < 0.05$.

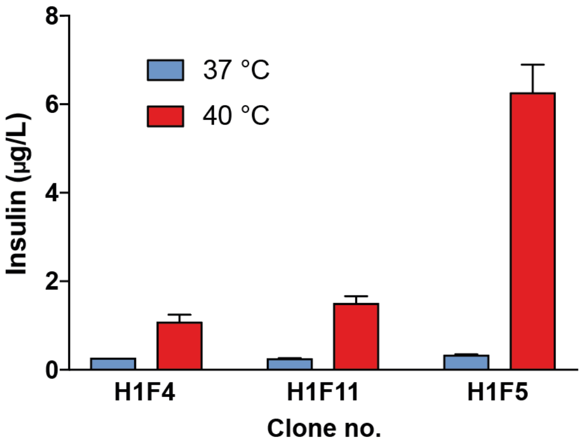

Supplementary Figure 10 | Selection of best-performing cell lines for establishing temperature-controlled insulin release. The best-performing monoclonal cell lines for heat-induced insulin secretion were selected from polyclonal populations stably expressing HEAT ($TlpA_{39}$-FFF-tTA) and mouse insulin (mINS) optimized for expression in HEK cells from an inducible $P_{hCMV*-1}$ promoter. Clone H1F5 was selected for further experiments. Data are shown as a bar graph of mean ± SD of n = 3 biologically independent samples, representative of three independent experiments.

Table S1. Plasmids designed and used in this study

Plasmid	Information and Design	Reference
pcDNA3. 1(+)	Mammalian expression vector. (P_{hCMV}-MCS-pA_{bGH})	Life Technologies, Carlsbad, CA
pSEAP2-Control	Constitutive P_{SV40}-driven SEAP reporter gene expression vector. (P_{SV40}-SEAP-pA_{SV40})	Clontech, Mountain View, CA
pDF145	In vitro ribozyme expression vector used as filler plasmid for equimolar transfections. (P_{T7}-SpAH-Env140ac)	Ausländer et al., 2016
pHY42	Constitutive mammalian OPN4 expression vector. (P_{hCMV}-OPN4-pA_{SV40})	Ye et al., 2011
pMM328	Constitutive mammalian SEAP expression vector. (P_{hPGK}-SEAP-pA_{bGH})	Chassin et al., 2019
pMM506	Constitutive mammalian tTA expression vector. (P_{hCMV}-tTA-pA_{bGH}; tTA, TetR-VP16)	Müller et al., 2017
pMM591	Constitutive mammalian rtTA expression vector. (P_{hCMV}-rtTA-pA_{bGH}; tTA, rTetR-VP16)	Chassin et al., 2019
pMX256	Sleeping Beauty-specific transposon encoding P_{NFAT5}-responsive SEAP and mINS expression as well as constitutive P_{RPBSA}-driven EGFP and ZeoR expression. (ITR-P_{NFAT5}-SEAP-P2A-mINS-pA_{SV40}:P_{RPBSA}-EGFP-P2A-ZeoR-pA_{bGH}-ITR)	Xie et al., 2016
pTS1017	Tetracycline-responsive SEAP expression vector. ($P_{hCMV*-1}$-SEAP-pA_{bGH}; $P_{hCMV*-1}$, O_{tetO7}-$P_{hCMVmin}$)	Scheller et al., 2020
pYL1	Mammalian reporter plasmid containing CRE, SRE, and NFAT binding sites upstream of a minimal promoter driving SEAP expression. ($O_{CRE-SRE-NFAT}$-$P_{hCMVmin}$-SEAP-pA_{SV40})	Liu et al., 2018
pWW124	γ-Butyrolactone (SCB1)-responsive SEAP expression vector. (P_{SPA}-SEAP-pA)	Weber et al., 2003
pcDNA3.0-FLAG-TRPV4	Constitutive mammalian FLAG-tagged TRPV4 expression vector. (P_{hCMV}-TRPV4-pA_{SV40})	Shukla et al., 2010 Addgene (no. 45751)
pFLAG-HSF1	Constitutive mammalian FLAG-tagged HSF1 expression vector. (P_{hCMV}-FLAG-HSF1-pA)	Wang et al., 2003 Addgene (no. 32537)
pTlpA39-Wasabi	Prokaryotic TlpA39-inducible mWasabi expression vector. (P_{TlpA39}-mWasabi-P_{LacI}-$TlpA_{39}$)	Piraner et al., 2017 Addgene (no. 86116)
pTRPV1	Constitutive mammalian TRPV1 expression vector. (P_{hCMV}-TRPV1-pA_{SV40})	Cao et al., 2013
pSB100x	Constitutive mammalian SB100X expression vector. (P_{hCMV}-SB100X-pA_{SV40})	Mátés et al., 2009 Addgene (no. 34879)
pMM590	Constitutive mammalian expression vector containing FFF. (P_{hCMV}-FFF-pA_{bGH})	Müller et al., Unpublished
pTS1022	Constitutive mammalian SEAP expression vector. (P_{hCMV}-SEAP-pA_{bGH})	Strittmatter et. al., Unpublished
pTS2336	Constitutive mammalian expression vector for the Nluc-mIgGFc. (P_{hCMV}-IgK-NLuc-mIgGFc-pA_{bGH})	Strittmatter et. al., Unpublished
pTS2338	Sleeping Beauty-specific transposon encoding a MCS as well as constitutive P_{RPBSA}-driven Ypet and PuroR expression. (ITR-MCS-pA_{bGH}:P_{RPBSA}-Ypet-P2A-PuroR-pA-ITR)	Strittmatter et. al., Unpublished
pTS2347	Sleeping Beauty-specific transposon encoding a MCS as well as constitutive P_{RPBSA}-driven mTagBFP2 and BlastR expression. (ITR-MCS-pA_{bGH}:P_{RPBSA}-mTagBFP2-P2A-BlastR-pA-ITR)	Strittmatter et. al., Unpublished

pSL3	Mammalian reporter plasmid containing CRE, SRE, and NFAT binding sites upstream of a minimal promoter driving EGFP expression. (O$_{\text{CRE-SRE-NFAT}}$-P$_{\text{hCMVmin}}$-EGFP-pA$_{\text{SV40}}$)	Lichtenstein et al., Unpublished
pAna115	Mammalian expression vector for TlpA-dependent SEAP expression. The oligonucleotides OAPT129 (5'-tcgagtttaatttgtttgttagttagtttatttgttggtttgtttgtgttataatattagagggtatataatggaagctcgacttccagg-3') and OAPT130 (5'-aattcctggaagtcgagcttccattatataccctctaatattataacacaaacaaaccaacaaataaactaactaacaaacaaattaaac-3') were annealed and cloned into pTS1022 restricted with *Xho*I/*Eco*RI. (O$_{\text{TlpA}}$-P$_{\text{min}}$-SEAP-pA$_{\text{bGH}}$)	This work
pAna117	Mammalian expression vector for constitutive expression of the TlpA-derived mammalian transcription factor TlpA$_{39}$-VP16. of a fusion protein consisting of TlpA$_{39}$ and VP16 transactivator domain. TlpA$_{39}$ was PCR-amplified from pTlpA39-Wasabi with OAPT131 (5'-taagcaactagtcgtccggcgacatacgaa-3') and OAPT132 (5'-taagcacgtacgcgcattctggccaccggt-3'), restricted with *Spe*I/*Bsi*WI and cloned into the corresponding sites (*Spe*I/*Bsi*WI) of pMM506. (P$_{\text{hCMV}}$-TlpA$_{39}$-VP16-pA$_{\text{bGH}}$)	This work
pAna126	Mammalian expression vector for constitutive expression of the TlpA-derived mammalian transrepressor TetR-TlpA$_{39}$. TlpA$_{39}$ was PCR-amplified from pTlpA39-Wasabi with OAPT139 (5'-taagcgtacgggtggttctggtggtagtggtggttctcgtccggcgacatacgaa-3') and OAPT138 (5'-taagactagtaccagaaccacccgcattctggccaccggt-3'), restricted with *Bsi*WI/*Spe*I and cloned into the corresponding sites (*Bsi*WI/*Nhe*I) of pMM506. (P$_{\text{hCMV}}$-TetR-TlpA$_{39}$-pA$_{\text{bGH}}$)	This work
pAB659	Constitutive mammalian expression vector encoding the protein thermometer TlpA$_{39}$-rtTA. rtTA was excised from pMM591 with *Spe*I/*Bam*HI and inserted into the compatible sites (*Nhe*I/*Bam*HI) of pBS659. (P$_{\text{hCMV}}$-TlpA$_{39}$-FFF-rtTA-pA$_{\text{bGH}}$)	This work
pBS1	Constitutive mammalian SEAP expression vector. SEAP was excised from pTS1017 with *EcoR*I/*Hind*III and inserted into the corresponding sites (*EcoR*I/*Hind*III) of EF1α-MCS. (P$_{\text{hEF1}\alpha}$-SEAP-pA$_{\text{bGH}}$)	This work
pBS628	Constitutive mammalian SEAP expression vector. SEAP was excised from pTS1017 with *EcoR*I/*Hind*III and inserted into the corresponding sites (*EcoR*I/*Hind*III) of pBS707. (P$_{\text{hCMV}}$-SEAP-pA$_{\text{bGH}}$)	This work
pBS659	Constitutive mammalian expression vector encoding the protein thermometer TlpA$_{39}$-FFF. TlpA was PCR-amplified using OBS103 (5'-accgaattcaccatgactagtcgtccggcgacatacgaaccagaacag-3') and OBS104 (5'-ggtggatccgctagccgcattctggccaccggtctgtttattgcctg-3') from pTlpA39-Wasabi, restricted with *Eco*RI/*Nhe*I and inserted in the compatible sites (*EcoR*I/*Spe*I) of pMM509. (P$_{\text{hCMV}}$-TlpA$_{39}$-FFF-pA$_{\text{bGH}}$)	This work
pBS667	Temperature-inducible mammalian SEAP expression vector. Complementary oligonucleotides OBS97 (5'-cgcgttactggaaagttcccgagtactggaaagttcccgagtactggaaagc-3') and OBS98 (5'-tcgagctttccagtactcgggaactttccagtactcgggaactttccagtaa-3') containing three tandem O$_{\text{hHSPA1A}}$ repeats of P$_{\text{hHSPA1A}}$ were annealed, phosphorylated and ligated into pTS1017 restricted with *Mlu*I/*Xho*I to result in pBS629. Subsequently, pBS629 was restricted with *Mlu*I/*Xho*I and OBS97/OBS98 was inserted. (O$_{\text{(hHSPA1A)6}}$-P$_{\text{hCMVmin}}$-SEAP-pA$_{\text{bGH}}$)	This work
pBS707	Constitutive mammalian expression vector encoding the protein thermometer HEAT (TlpA$_{39}$-FFF-tTA). tTA was excised from pMM506 with *Spe*I/*Hin*dIII and inserted into the compatible sites (*Nhe*I/*Hin*dIII) of pBS659. (P$_{\text{hCMV}}$-TlpA$_{39}$-FFF-tTA-pA$_{\text{bGH}}$).	This work
pBS717	Tetracycline-inducible mammalian NLuc-mIgGFc expression vector. NLuc-mIgGFc excised was from pTS2336 with *Spe*I/*Hin*dIII and inserted into the corresponding sites (*Spe*I/*Hin*dIII) of pTS1017. (P$_{\text{hCMV*-1}}$-IgK-NLuc-mIgGFc-pA$_{\text{bGH}}$)	This work
pBS736	Constitutive mammalian expression vector encoding the protein thermometer HEAT (TlpA$_{39}$-FFF-tTA). TlpA$_{39}$-FFF-tTA was excised from pBS707 with *Eco*RI/*Hin*dIII and inserted into the corresponding sites (*Eco*RI/*Hin*dIII) of P$_{\text{hEF1}\alpha}$-SEAP. (P$_{\text{hEF1}\alpha}$-TlpA$_{39}$-FFF-tTA-pA$_{\text{bGH}}$)	This work
pBS786	Sleeping Beauty-specific transposon encoding P$_{\text{hEF1}\alpha}$-driven HEAT (TlpA$_{39}$-FFF-tTA) expression as well as constitutive P$_{\text{RPBSA}}$-driven mTagBFP2 and BlastR expression. P$_{\text{hEF1}\alpha}$-TlpA$_{39}$-FFF-tTA was excised from pBS736 with *Bgl*II/*Hin*dIII and	This work

	inserted into the corresponding sites (*Bgl*II/*Hin*dIII) of pTS2347. (ITR-P$_{hEF1\alpha}$-TlpA$_{39}$-FFF-tTA-pA$_{bGH}$:P$_{RPBSA}$-mTagBFP2-2A-BlastR-pA$_{bGH}$-ITR)	
pBS825	Constitutive mammalian expression vector encoding the protein thermometer HEAT (TlpA$_{39}$-FFF-tTA). TlpA$_{39}$-FFF-tTA was excised from pBS707 with *Eco*RI/*Hin*dIII and inserted into the corresponding sites (*Eco*RI/*Hin*dIII) of P$_{PGK}$-SEAP. (P$_{hPGK}$-TlpA$_{39}$-FFF-tTA-pA$_{bGH}$)	This work
pBS828	Sleeping Beauty-specific transposon encoding P$_{hCMV*-1}$-driven SEAP and mINS expression as well as constitutive P$_{RPBSA}$-driven YPet and PuroR expression. P$_{hCMV*-1}$ was excised from pTS1017 with *Mlu*I/*Eco*RI and inserted into the corresponding sites (*Mlu*I/*Eco*RI) of pMX256. (ITR-$_{PhCMV*-1}$-SEAP-P2A-mINS-pA$_{bGH}$:P$_{PRPBSA}$-EGFP-P2A-ZeoR-pA$_{bGH}$-ITR)	This work
pBS830	Sleeping Beauty-specific transposon encoding P$_{hPGK}$-driven HEAT (TlpA$_{39}$-FFF-tTA) as well as constitutive P$_{RPBSA}$-driven YPet and PuroR expression. P$_{hEF1\alpha}$-TlpA$_{39}$-FFF-tTA was excised from pBS736 with *Bgl*II/*Hin*dIII and inserted into the corresponding sites (*Bgl*II/*Hin*dIII) of pTS2338. (ITR-P$_{hPGK}$-TlpA$_{39}$-FFF-tTA-pA$_{bGH}$:P$_{RPBSA}$-YPet-P2A-PuroR-pA$_{bGH}$-ITR)	This work
pBS876	Sleeping Beauty-specific transposon encoding P$_{hCMV*-1}$-driven NLuc-mIgGFc expression as well as constitutive P$_{RPBSA}$-driven Zeocin and mRuby2 expression. P$_{hCMV*-1}$ was excised from pBS717 with *Mlu*I/*Eco*RI and inserted into the corresponding sites (*Mlu*I/*Eco*RI) of pTS2349. (ITR-$_{PhCMV*-1}$- NLuc-mIgGFc -pA$_{bGH}$:P$_{PRPBSA}$-ZeoR-P2A-mRuby2-pA-ITR)	This work
pKK60	Synthetic mammalian heat shock promoter-driven SEAP expression vector. P$_{hHSP70B'}$ was PCR-amplified from HEK-293 using oligonucleotides OKK145 (5'-attagacgtcggggatacaaacagtggggag-3') and OKK146 (5'-attacctgcaggtgctcagctccgcttccac-3'), restricted with *Aat*II/*Sbf*I and cloned into the corresponding sites (*Aat*II/*Sbf*I) of pWW124. (P$_{hHSP70B'}$-P$_{hCMVmin}$-SEAP-pA$_{SV40}$)	This work
pLeo1402	Mammalian reporter plasmid containing CRE, SRE, and NFAT binding sites upstream of a minimal promoter driving Nluc-mIgGFc expression. Nluc-mIgGFc was excised from pBS717 with *Spe*I/*Hin*dIII and inserted into the corresponding sites (*Spe*I/*Hin*dIII) of pSL3. (O$_{CRE-SRE-}$NFAT-P$_{hCMVmin}$-NLuc-mIgGFc-pA$_{bGH}$)	This work

Abbreviations: **BlastR**, blasticidin S resistance gene; **CRE**, cyclic adenosine monophosphate response element; **EGFP**, enhanced green-fluorescent protein; **FFF**, trimeric tandem repeat of VP16-derived F-type core transactivation domain; **FLAG**, epitope tag for recombinant protein purification; **GPCR**, G-protein-coupled receptor; **HEAT**, human enhanced gene activation thermometer; **HSF1** human heat shock factor 1; **IgK** light-chain kappa-derived secretion signal; **ITR** inverted terminal repeats of SB100X; **MCS**, multiple cloning site; **mIgGFc** murine immunoglobulin gamma heavy chain constant region; **mINS**, optimized insulin variant for expression in HEK-293 cells; **mTagBFP2** modified blue-fluorescent protein variant; **mWasabi**, modified green-fluorescent variant of the *Clavularia* cyan-fluorescent protein; **NFAT**, nuclear factor of activated T-cells; **Nluc**, nanoluc luciferase reporter gene; **O$_{(hHSPA1A)6}$**, operator containing six tandem repeats of O$_{HSPA1A}$; **O$_{HSPA1A}$**, operator of the heat shock protein family A member 1A promoter; **O$_{tetO7}$**, heptameric TetR-specific operator; **OPN4**, blue light-sensitive GPCR melanopsin; **P2A** picornavirus-derived ribosome skipping sequence optimized for bicistronic expression in mammalian cell; **pA$_{bGH}$**, polyadenylation signal from the bovine growth hormone; **pA$_{SV40}$**, polyadenylation signal from simian virus 40; **PCR** polymerase chain reaction; **P$_{hEF1\alpha}$**, human elongation factor 1-alpha promoter; **P$_{hCMV}$**, human cytomegalovirus immediate early promoter; **P$_{hCMVmin}$**, minimal version of P$_{hCMV}$; **P$_{hCMV*-1}$**, tetracycline-responsive promoter; **P$_{hPGK}$**, human 3-phosphoglycerate kinase promoter; **P$_{hHSP70B'}$**, human heat shock protein 7 kDa member 6 promoter; **P$_{LacI}$**, promoter of the *Escherichia coli* LacI repressor; **P$_{NFAT5}$**, promoter containing pentameric tandem NFAT operator sequences; **P$_{RPBSA}$**, strong synthetic promoter; **P$_{SPA}$**, SPA-dependent promoter; **P$_{T7}$**, phage T7 RNA polymerase promoter; **P$_{TlpA}$**, TlpA promoter; **PuroR**, puromycin resistance gene; **SB100X** optimized Sleeping

Beauty transposase; **SCB1**, 2-(1'hydroxy-6-methylheptyl)-3-(hydroxymethyl)butanolide; **SEAP**, human placental secreted alkaline phosphatase; **SPA**, *Streptomyces pristinaespiralis*-derived transcription factor (SpbR-VP16); **SpAH-Env140ac**, engineered hammerhead ribozyme with high stem III thermodynamic stability; **SpbR**, *Streptomyces pristinaespiralis* butyrolactone-dependent repressor; **SRE**, serum response element; **$TlpA_{39}$** modified version of TlpA; **TlpA**, a temperature-sensitive repressor of *Salmonella typhimurium*; **TetR** *Escherichia coli Tn*10-derived tetracycline-dependent repressor; **tTA**, tetracycline-dependent transactivator (TetR-VP16); **rTetR**, reverse TetR; **rtTA**, reverse tetracycline-dependent transactivator (rTetR-VP16); **TRPV4**, transient receptor potential vanilloid 4; **TRPV1**, transient receptor potential vanilloid 1; **VP16**, *Herpes simplex* virus-derived transactivation domain; **Ypet**, modified yellow-fluorescent protein; **ZeoR**, zeocin resistance gene.

Oligonucleotides: sequences specific for restriction endonucleases are underlined.

References

Ausländer S, Fuchs D, Hürlemann S, Ausländer D, Fussenegger M. Engineering a ribozyme cleavage-induced split fluorescent aptamer complementation assay. Nucleic Acids Res. 2016, 44: e94.

Cao E, Liao M, Cheng Y, Julius D. TRPV1 structures in distinct conformations reveal activation mechanisms. Nature 2013, 504:113-118.

Chassin H, Müller M, Tigges M, Scheller L, Lang M, Fussenegger M. A modular degron library for synthetic circuits in mammalian cells. Nat Commun. 2019 May 1;10(1):2013.

Liu Y, Charpin-El Hamri G, Ye H, Fussenegger M. A synthetic free fatty acid regulated transgene switch in mammalian cells and mice. Nucleic Acids Res. 2018, 46: 9864-9874.

Mátés L, Chuah MK, Belay E, Jerchow B, Manoj N, Acosta-Sanchez A, Grzela DP, Schmitt A, Becker K, Matrai J, Ma L, Samara-Kuko E, Gysemans C, Pryputniewicz D, Miskey C, Fletcher B, VandenDriessche T, Ivics Z, Izsvák Z. Molecular evolution of a novel hyperactive Sleeping Beauty transposase enables robust stable gene transfer in vertebrates. Nat Genet. 2009, 41: 753-761.

Müller M, Ausländer S, Spinnler A, Ausländer D, Sikorski J, Folcher M, Fussenegger M. Designed cell consortia as fragrance-programmable analog-to-digital converters. Nat Chem Biol. 2017 Mar;13(3):309-316.

Piraner DI, Abedi MH, Moser BA, Lee-Gosselin A, Shapiro MG. Tunable thermal bioswitches for in vivo control of microbial therapeutics. Nat. Chem. Biol. 2017, 13: 75-80.

Scheller L, Schmollack M, Bertschi A, Mansouri M, Saxena P, Fussenegger M. Phosphoregulated orthogonal signal transduction in mammalian cells. Nat. Commun. 2020, 11: 3085.

Shukla AK, Kim J, Ahn S, Xiao K, Shenoy SK, Liedtke W, Lefkowitz RJ. Arresting a transient receptor potential (TRP) channel: beta-arrestin 1 mediates ubiquitination and functional down-regulation of TRPV4. J. Biol. Chem. 2010, 285: 30115-30125.

Wang X, Grammatikakis N, Siganou A, Calderwood SK. Regulation of molecular chaperone gene transcription involves the serine phosphorylation, 14-3-3 epsilon binding, and cytoplasmic sequestration of heat shock factor 1. Mol. Cell Biol. 2003, 23: 6013-6026.

Weber W, Schoenmakers R, Spielmann M, Daoud-El Baba M, Folcher M, Keller B, Weber CC, Link N, van de Wetering P, Heinzen C, Jolivet B, Séquin U, Aubel D, Thompson CJ, Fussenegger M. Streptomyces-derived quorum-sensing systems engineered for adjustable transgene expression in mammalian cells and mice. Nucleic Acids Res. 2003, 31: e71.

Xie M, Ye H, Wang H, Charpin-El Hamri G, Lormeau C, Saxena P, Stelling J, Fussenegger M. β-cell-mimetic designer cells provide closed-loop glycemic control. Science 2016, 354: 1296-1301.

Ye H, Daoud-El Baba M, Peng RW, Fussenegger M. A synthetic optogenetic transcription device enhances blood-glucose homeostasis in mice. Science 2011, 332: 1565-1568.
